# Microsaccadic Eye Movements but not Pupillary Dilation Response Characterizes the Crossmodal Freezing Effect

**DOI:** 10.1093/texcom/tgaa072

**Published:** 2020-09-30

**Authors:** Lihan Chen, Hsin-I Liao

**Affiliations:** Department of Brain and Cognitive Sciences, Schools of Psychological and Cognitive Sciences, Peking University, Beijing, 100871, China; Beijing Key Laboratory of Behavior and Mental Health, Peking University, Beijing, 100871, China; Key Laboratory of Machine Perception (Ministry of Education), Peking University, Beijing, 100871, China; NTT Communication Science Laboratories, NTT Corporation, Atsugi, Kanagawa, 243-0198, Japan

**Keywords:** audiovisual integration, microsaccades, perceptual organization, Ternus display

## Abstract

In typical spatial orienting tasks, the perception of crossmodal (e.g., audiovisual) stimuli evokes greater pupil dilation and microsaccade inhibition than unisensory stimuli (e.g., visual). The characteristic pupil dilation and microsaccade inhibition has been observed in response to “salient” events/stimuli. Although the “saliency” account is appealing in the spatial domain, whether this occurs in the temporal context remains largely unknown. Here, in a brief temporal scale (within 1 s) and with the working mechanism of involuntary temporal attention, we investigated how eye metric characteristics reflect the temporal dynamics of perceptual organization, with and without multisensory integration. We adopted the crossmodal freezing paradigm using the classical Ternus apparent motion. Results showed that synchronous beeps biased the perceptual report for group motion and triggered the prolonged sound-induced oculomotor inhibition (OMI), whereas the sound-induced OMI was not obvious in a crossmodal task-free scenario (visual localization without audiovisual integration). A general pupil dilation response was observed in the presence of sounds in both visual Ternus motion categorization and visual localization tasks. This study provides the first empirical account of crossmodal integration by capturing microsaccades within a brief temporal scale; OMI but not pupillary dilation response characterizes task-specific audiovisual integration (shown by the crossmodal freezing effect).

## Introduction

Most events in our daily life consist of perceptual inputs from more than 1 modality. According to the principle of functional appropriateness and precision associated with each sensory modality (Welch and Warren 1980), inputs from different sensory modalities integrate and influence each other to maximize the performance of the task at hand. Recent behavioral and neurophysiological evidence has shown the inverse effectiveness principle in multisensory integration: in adverse conditions the perceptual discrimination of target events/stimuli will benefit from inputs from another sensory modality (Holmes 2009; Crosse et al. 2016; Hou et al. 2019). This benefit—known as multisensory gain—has been observed in the crossmodal freezing effect: an abrupt sound affects the processing of a rapidly presented visual stimulus, which is also known as the “freezing phenomenon.” When subjects are shown a rapidly changing visual display, an abrupt sound “freezes” the display with which the sound is synchronized. Perceptually, it appears as though the display is brighter or shown for a longer time (Vroomen and de Gelder 2000).

The “freezing phenomenon” has been recently robustly observed in a classic visual apparent motion Ternus display. The display triggers mutually exclusive bistable apparent motion percepts of either element (retinotopic) or group (nonretinotopic) motion, depending on the perceived time interval between 2 fast/transient visual frames (Ternus 1926; Harrar and Harris 2007; Chen et al. 2010). With concurrent auditory inputs, observers reported a more dominant percept of “group motion” during a Ternus display, in which the interval between 2 visual frames paired with beeps had been perceived as longer than when there were no paired auditory inputs (Wearden, et al. 1998; Shi et al. 2010; Chen et al. 2018). During a perceptual experience, concurrent auditory inputs render the individual frames more distinctly, resembling a similar “freezing” role as observed by Vroomen and de Gelder (2000). In visual Ternus displays, the illusory prolonged time interval led to more frequent reports of “group motion.” Neuroimaging evidence has shown that human middle temporal complex (hMT+) may be the first visual area that encodes nonretinotopic percepts of the Ternus apparent motion, whereas the blood-oxygen-level dependent activations in V1, V2, and V3 reflect the retinotopic properties of the Ternus display (Thunell et al. 2016). Computational modeling work has developed a neural network model of motion segmentation by the visual cortex, by outlining a Motion Boundary Contour System (Grossberg 1989).

The parsing of visual Ternus motion requires solving the problem of motion correspondence between 2 visual frames (Hein and Cavanagh 2012). The visual elements in a visual frame will favor a within-frame (spatial) perceptual grouping, whereas the visual elements between 2 frames will trigger a between-frames (temporal) grouping (Kramer and Yantis 1997). Within the short temporal scale (around 300 ms), the within-frame grouping corresponds to retinotopic processing, whereas the between-frames grouping mainly adopts nonretinotopic processing (Thunell et al. 2016; Lauffs et al. 2019). This competition between retinotopic and nonretinotopic processing makes the percept of visual apparent motion less stable.

With auditory inputs, there is perceptual competition (assimilation) between auditory signals and parsing of visual motion. Concurrent auditory inputs have shown to stabilize nonstable visual motion percepts (Freeman and Driver 2008) and to counteract the otherwise ambiguous percept of visual Ternus apparent motion (Shi et al. 2010; Chen et al. 2018). In the default state, people rely on retinotopic processing to follow transient visual stimuli (Amit et al. 2019; Betta and Turatto 2006; Boehnke and Munoz 2008; Boi et al. 2009; Brien et al. 2009; Dankner et al. 2017; Findlay 1974; Fried et al. 2014; Hafed et al. 2011; Olmos-Solis et al. 2017; Pastukhov and Braun 2010a). On the other hand, human and primates usually had large and frequent fixational eye movements which may lead to nonretinotopic processing. To keep our perceptual world stable, it is surmised that auditory inputs engage predominantly nonretinotopic processing by freezing the individual visual stimuli (Ternus frames), which is reflected in more frequent reports of group motion when participants observe a visual Ternus display with concurrent sounds (Kong et al. 2014; Braga et al. 2017). Therefore, nonretinotopic processing fills in the gaps between the retinal images of each fixational, yet unstable, eye movements during individual Ternus frames (Otaki et al. 2014), which can lead to microsaccade inhibition (Wang et al. 2017; Amit et al. 2019; Denison et al. 2019).

Perceptual classification of the visual Ternus display (apparent motion), mobilizes the process of oculomotor planning and execution. Eye-tracking techniques have been a valuable tool for capturing the temporal dynamics of audiovisual integration, allowing close investigation of the multisensory integration during the freezing effect. However, this approach has its limitations; Ternus motion takes place in a very narrow spatial range (within 2 degrees in our case), where normal/regular saccades are rarely observed. On the other hand, microsaccades may reveal finer detail during Ternus presentations, though surprisingly, empirical evidence is not well documented.

Microsaccades, as 1 type of fixational eye movement, can preserve vision by preventing perceptual fading (Zuber and Stark 1966; Beeler 1967; Engbert and Mergenthaler 2006; Martinez-Conde et al. 2006, 2009, 2013; Hafed and Krauzlis 2010, 2012; Hafed and Ignashchenkova 2013; Park et al. 2019). Furthermore, microsaccades can drive typical illusory motions, such as in the Enigma illusion (MonWilliams and Wann 1996; Troncoso et al. 2008). In contrast, the perturbation of microsaccade rate (i.e., suppression) can reduce visual cortex excitability for detecting target events, such as the second target (T2) in the attentional blink task (Pastukhov et al. 2013; Roberts et al. 2019). In the auditory domain, the rate of microsaccades (the fastest component of miniature eye movements), is transiently modulated after auditory stimulation and is used to categorize sound identity (80–100 ms after the onset of target beeps) before sound representation is established (N1 component of the auditory evoked potential) (Widmann et al. 2014), favoring a predictive coding model (Friston 2005, 2010). Although it has been shown that microsaccades (MS) characterize visual and auditory perception individually, it is unclear how they might play an important role in audio-visual integration, as a way to stabilize the perceptual environment. Recent evidence has shown that the pupil dilates as a response to the appearance of salient auditory and/or visual stimuli, which has been presumed to be based on the neural activation of the superior colliculus (Wang et al. 2014, 2015, 2016, 2017; Liao et al. 2016a; Liao et al. 2016b). Although the superior colliculus is known to serve as a multisensory integration hub (Wallace et al. 1996, 1998; Wallace and Stein 1997; Ursino et al. 2009), the pupil dilation response (PDR) may characterize the crossmodal freezing effect and reveal inherent temporal dynamics.

With the 2 potential candidates of MS and pupil size, we aimed to discover how the crossmodal integration as well as its brief temporal course could be well described with eye movement metrics. To this end, in the present study we used a Ternus display with concurrent beeps. Enhanced percepts (more frequent reports) of “group motion” can be acquired by boosting the “salience” of each individual visual Ternus frame with the use of auditory inputs, alongside the pupillary dilation response (Wang et al. 2017). Crossmodal integration of audiovisual events can be considered as an attentionally demanding process (Watanabe and Shimojo 2001), which may suppress MS more effectively (Engbert and Kliegl 2003) than less attentionally demanding tasks (i.e., unimodal visual Ternus apparent motion). This makes the otherwise ambiguous visual percepts more stable and mobilizes mainly nonretinotopic processing to favor a dominant percept of “group motion.” We expect to see subsequent increased frequency (“rebounds”) of microsaccades, which we presume reflects a relaxation of attention while following the targets/crossmodal events (Rolfs et al. 2008a; Hafed and Krauzlis 2010; Pastukhov and Braun 2010b). Furthermore, we anticipate the delay of the rebounds to be prolonged in the crossmodal compared with the unimodal condition.

We found that the inputs of concurrent paired beeps counteracted the otherwise unstable/ambiguous retinal slip of visual Ternus frames, with observable oculomotor inhibitions (OMI) and reduced MS rates). The MS rebounds were slower when the visual Ternus frames were presented with concurrent paired beeps than without beeps. The control experiment, which aimed to examine whether the sound per se, without integrating with visual input, modulates MS, did not show this difference in a separate task where audio-visual integration was not required (e.g., a visual localization task). In contrast, we found that although pupils dilated in response to beeps, the amount of dilation was similar regardless of whether or not audio-visual integration was required. Overall, the results suggest that MSs with a temporal attending process characterizes the crossmodal freezing effect.

## Materials and Methods

### Subjects

A total of 69 participants (17, 16, 18, and 18, in Experiments 1–4, respectively), ages ranging from 19 to 40 years, participated in the 4 experiments. All participants had normal or corrected-to-normal vision and reported normal hearing. Among the participants, 6 subjects took part in Experiments 1 and 4, 1 subject took part in both Experiments 1 and 2, and 1 participant took part in 3 experiments (Experiments 2–4). We predicted a medium effect size (φ = 0.40) for our experimental design. To ensure adequate power, we performed a power calculation in G*power 3 (Faul et al. 2009) with F tests, multivariate analysis of variance (ANOVA) repeated measures, within-between interaction, which determined that with a significance level (α) of 0.05, the sample size needed to achieve a power level of 1 − β = 0.80 was 73 individuals for the 4 experiments.

All experiments were performed in compliance with the institutional guidelines set by the Academic Affairs Committee, School of Psychological and Cognitive Sciences, Peking University, China, and according to the Helsinki Declaration of 1975 concerning human and animal rights. All participants provided written informed consent according to institutional guidelines and were reimbursed for their time with 50 CNY/h.

### Apparatus and Stimuli

The experiments were conducted in a dimly lit (luminance: 0.09 cd/m^2^) testing room. Visual stimuli were presented at the center of a 22-inch cathode-ray tube monitor (FD 225P) at a screen resolution of 1024 × 768 pixels and a refresh rate of 100 Hz. The viewing distance was 57 cm, which was maintained using a chin rest.

**Figure 1:**
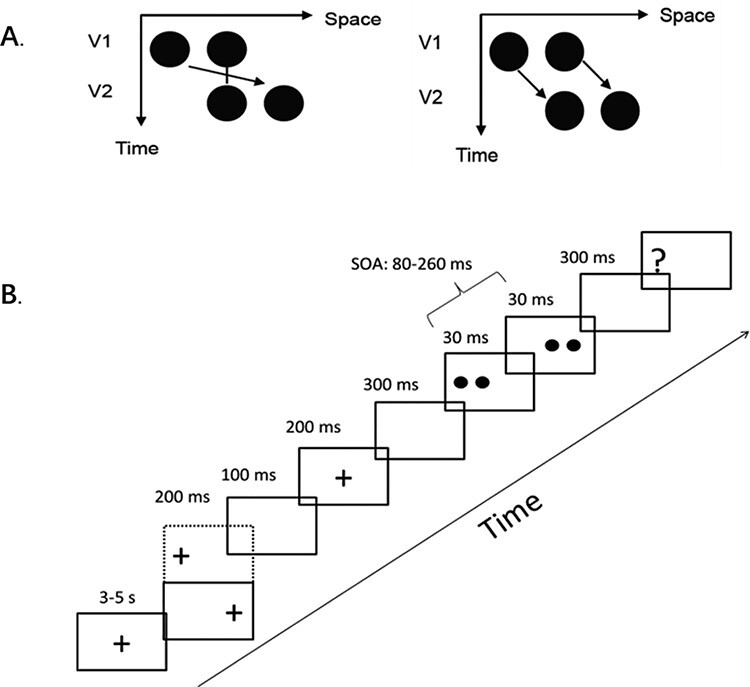
Ternus display and stimulus configurations. (*A*) Two alternative motion percepts of the Ternus display. Left: “element” motion for short ISI, where the middle dot is perceived as static, whereas the outer dots are perceived to move from 1 side to the other. Right: “group” motion for long ISIs, where the 2 dots are perceived as moving in tandem. (*B*) Example trial for Experiments 1–3.

The Ternus display consisted of 2 stimulus frames, each containing 2 black discs (10.24 cd/m^2^; disk diameter and separation between discs were 1.6° and 3° of visual angle, respectively), which were presented on a gray background (16.3 cd/m^2^). Both frames shared 1 element location at the center of the monitor while containing 2 further elements located at horizontally opposite positions relative to the center (see Fig. 1*A*). Each frame was presented for 30 ms; the interstimulus interval (ISI) between both frames was randomly selected from a range of 50–230 ms, with a step size of 30 ms. A blank screen (with the same gray background) was present during the ISI.

Mono sound beeps (1000 Hz, 65 dB, and 30 ms duration) were generated and delivered via an M-Audio card (Delta 1010) to a headset (Philips, SHM1900) worn by the participant. To ensure accurate timing of auditory and visual stimuli, the duration of the visual stimuli and the synchronization between the auditory and visual stimuli were controlled via the vertical synchronization pulses of the monitor. The experimental program was written using Matlab (Mathworks Inc.) and Psychophysics Toolbox (Brainard 1997; Pelli 1997; Kleiner et al. 2007).

## Experimental Design

### Practice

Before the formal experiment, participants were trained to become familiar with the Ternus displays. When the spatial configuration is fixed, observers typically report 2 distinct percepts (element motion and group motion) depending on the ISI. Short ISIs usually give rise to the percept of element motion, where the outer dots are perceived as moving, whereas the central dot appears to remain static or flashing. In contrast, long ISIs give rise to the perception of group motion, whereby the 2 dots are perceived to be moving together as a group. During the practice block, only ISIs of 50 ms (typical “element motion”) and 260 ms (typical “group motion”) were used. Participants were asked to discriminate whether the apparent motion they saw was an element motion or group motion, by pressing the left or the right mouse button, respectively. They pressed the left button to indicate the “element motion” response and right button for “group motion” response. When an incorrect response was registered, immediate feedback appeared on the screen that showed the correct response (i.e., element or group motion). This practice session continued until the participant reached a mean accuracy of 95%. All participants achieved this within 120 trials. After the practice, participants went through the formal experiment with the ISI varying between 50 and 230 ms. They performed the same apparent motion discrimination task but were not provided with feedback.

#### Experiment 1: Fully Randomized Ternus Task

The trial started with the presentation of a central fixation cross with a size of 1° for 3–5 s. Then, the fixation appeared immediately either on the left or right side of the screen for 200 ms, with an eccentricity of 9.7°. After the presentation of a blank screen for 100 ms, the fixation cross-returned to the center of the screen, where it remained for another 200 ms. After 300 ms of a blank presentation, the Ternus frames were presented, which were either synchronized with the 2 auditory beeps or without the beeps. After the second Ternus frame, a blank screen was presented for 300 ms, followed by a screen with a question mark (font *Arial*, size 32, either on the left or right of the screen, with a 10.3° eccentricity to the central fixation). Participants were asked to make a forced-choice of 2 alternatives, indicating the type of perceived motion (element or group motion). The question mark disappeared when the participants made a response. As mentioned earlier, the ISI between the Ternus frames (i.e., duration between the offset of the first Ternus frame and the onset of the second Ternus frame) was randomly selected from 1 of the following 7 intervals: 50, 80, 110, 140, 170, 200, and 230 ms, during which a blank screen was presented. This procedure led to the stimulus onset asynchrony (SOA) of the Ternus frames (i.e., duration between the onsets of the 2 Ternus visual frames) to be the following intervals: 80, 110, 170, 140, 200, 230, and 260 ms. There were 24 trials for each level of SOA, which were counterbalanced between left- and rightward apparent motion, no-sound, and sound conditions. The order in which trials were presented was randomized for each participant. Participants performed a total of 336 trials, divided into 2 blocks of 168 trials each. Throughout the experiments, participants were required to fixate the fixation cross and make saccades whenever the fixation cross-moved (Fig. 1*B*). This procedure was used to promote/generate a new fixation immediately before the Ternus frames, to decrease the chance for participants to blink and/or make saccades during the Ternus frames and thus allowing better eye movement data acquisition (see eye movement recording and data analyses for details).

#### Experiment 2: Ternus Motion with Block-Sound Conditions

The stimuli configuration and timelines were identical to Experiment 1, except that the sound conditions (with or without beeps) were separated into different blocks. Specifically, the experimental trials were separated into 4 blocks: 2 blocks consisted of the Ternus display without tones, whereas the other 2 blocks had synchronously paired beeps. Each block contained 84 trials. The level of SOA and the left- or rightward apparent motion were counterbalanced and presented in a randomized order. The order of blocks for baseline (no-sound) and sound conditions was randomized using the Latin square protocol.

#### Experiment 3: Ternus Motion with Block-SOA Conditions

The stimulus configuration and timelines were identical to Experiment 1, except that the 7 levels of SOA conditions were conducted in separate blocks each containing 168 trials. However, the SOAs in a given block were fixed. There were 12 consecutive trials for each level of SOA and left- and rightward apparent motion was counterbalanced.

#### Experiment 4: Localization of Visual Stimulus (Control Test)

Experiment 4 was the control experiment. The trial structure and time parameters were identical to Experiment 1, except that the critical Ternus frames were replaced by a blank screen (with the same luminance as the background). Upon seeing the question mark, participants were required to discriminate whether the question mark appeared at a left or right location by clicking the left mouse button or right mouse button, respectively, as quickly and as accurately as possible.

### Behavioral Data Analyses

For Experiments 1–3, the proportions of the group-motion responses across 7 intervals were fitted to the psychometric curve using a logistic function (see Shi et al. 2010). The transitional threshold, which is the points of subjective equality (PSE) at which the participant was likely to report both motion percepts equally, was calculated by estimating 50% of the reporting of group motion on the fitted curve. The just noticeable difference (JND), which is an indicator of the sensitivity of apparent motion discrimination, was calculated as 50% of the difference between the lower (25%) and upper (75%) bounds of the thresholds of the psychometric curve. For Experiment 4, we analyzed the reaction time (RT) of visual localizations of the target question mark.

### Eye Movement Recording and Data Analyses

Eye movement data of the right eye were acquired noninvasively by a video-based infrared camera (Eyelink 1000, SR Research), with a 500-Hz sampling rate. We extracted 3 eye-tracking measures: gaze duration, OMI (i.e., the MS rate), and pupil diameter change. These 3 measures aimed to look into different eye characteristics to see how sounds affect crossmodal integration in different time scales. Gaze duration analysis aimed to examine how the fixation/gaze could be maintained during the Ternus display. OMI and pupil diameter change were used to examine these 2 eye characteristics change over time after the stimulus presentation.

For the analysis of gaze duration, we examined the length of a fixation during the presentations of Ternus visual frames as a function of SOAs and sound conditions. Specifically, each fixation was identified by the Eyelink data viewer software and exported into the nondelimited American Standard Code for Information Interchange (ASCII) format for further analysis (using the data transform function, edf2asc, provided by SR Research). The fixation that was held for the whole Ternus motion presentation was identified as the “gaze” for each trial. That is, the fixation that occurred before the onset of the first Ternus frame and ended after the offset of the second Ternus frame. Data were excluded if a response was registered during the gaze.

For OMI analysis, MS rate changes were time-locked to onsets of both Ternus frames, respectively, to investigate the time course of the sound effect upon visual motion “categorization” within a short time window (~1 ). Here we defined microsaccades as “involuntary saccades while the subject is attempting to fixate” (Martinez-Conde 2006), which could be observed during various viewing tasks including exploration and visual search (Otero-Millan et al. 2008). Specifically, MS were detected using velocity thresholds (Engbert and Kliegl 2003). We used a threshold of λ = 8 times the median-based standard deviation for the horizontal component within each session. The extracted MS were excluded if their duration was shorter than 3 ms, larger than 110 ms, or if the interval to the previous microsaccade was shorter than 20 ms. Supplementary Figure 1 shows the correlation between the microsaccade amplitude and peak velocity of all the detected microsaccades in the 3 main experiments (*r* = 0.72, *P* < 0.001). As illustrated in Supplementary Figure 1, the majority of the detected MS was within 1.5 degree of visual angle, which was considered quite small, compared with the normal saccades which could be over 10 degrees (Bahill et al. 1975). To compute the OMI rate, the sum of MS was normalized by the number of trials and the sampling rate for each condition and each participant. Because it is assumed that a maximum exists, which would degrade the impact of a MS on the MS rate before the respective time point, a causal smoothing kernel ω(τ) = α^2^ τ exp(−ατ) was applied with a decay parameter of α = 1/20 ms (Dayan and Abbott 2001; Rolfs et al. 2008b). The OMI rate was averaged across participants but separated by each condition.

For the pupillary response analysis, the pupil diameter data were time-locked to the onset of the first Ternus visual frame. Data during blinks were linearly interpolated. The Eyelink system outputted arbitrary units [au], which ranged from 175 to 11 832 (in the current study) and represented pupil size. To compare the size across conditions and participants, pupil size data were normalized by the mean and variance of the data recorded for each session and also baseline corrected by the mean of the data 0.1 s before the first visual frame presentation.

### Statistical Analysis

For the averaged results, data from Experiments 1–3 were collapsed and mean PSE and JND were subjected to a repeated measures ANOVA, with sound condition (baseline/no-sound vs. sound) as the within-subject factor. Mean reaction times (for Experiment 4) and gaze durations (for all experiments) were subjected to a repeated-measures ANOVA with sound condition (sound, no-sound) and the 7-levels of SOAs as within-subject factors. For OMI and pupillary responses, nonparametric cluster-based permutation tests (Maris and Oostenveld 2007) were performed to identify differences between the sound and baseline (no-sound) conditions for both the OMI rate and pupillary responses. This was carried out for SOAs collapsed across all levels as well as for each SOA condition. Cluster-based analyses were computed using the Fieldtrip MATLAB toolbox (Oostenveld et al. 2011) with 1000 iterations and an α-level of 0.05. For between experiment comparisons of MS rates and pupil diameter change, we used time bins of 250-ms time windows between 0 and 1 s in reference to the first/second visual frame. These data were subjected to a mixed-model ANOVA with time window and sound condition as the within-subject factors and experiment as the between-subjects factor.

All data analyses were implemented in Matlab 2018b (Mathworks Inc.) and IBM SPSS statistics (version 20).

## Results

### Psychometric Findings and Microsaccades

The mean PSE [± standard error (SE)] for Experiment 1 (“fully randomized trials”), Experiment 2 (“block-sound”), and Experiment 3 (“block-SOA”) were 150.0 (±5.7) ms, 140.3 (±5.9) ms, and 130.3 (±5.5) ms, respectively. The main effect of experiment was marginally significant [*F*(2,48) = 3.079, *P* = 0.055, *η*^2^ = 0.114]. PSE in Experiment 1 was slightly higher than in Experiment 3 (*P* = 0.050). No differences in PSEs were found between Experiment 1 and Experiment 2 (*P* = 0.725), or between Experiment 2 and Experiment 3 (*P* = 0.663). The interaction between sound condition and experiment was significant [*F*(2,48) = 13.044, *P* < 0.001, *η*^2^ = 0.352]. The simple effects analysis showed that in the baseline condition, PSE was largest in Experiment 1 (214.2 ± 8.9 ms), *Ps* < 0.01, whereas no difference was found between Experiment 2 (160.3 ± 9.2 ms) and Experiment 3(165.8 ± 8.6 ms), *P* = 1. In the sound condition, PSE was smaller in Experiment 1 (85.8 ± 7.8 ms) compared with Experiment 2 (120.2 ± 8.0 ms; *P* < 0.001). The PSE was slightly smaller in Experiment 3 (94.7 ± 7.6 ms) compared with Experiment 2 (120.2 ± 8.0 ms; *P* = 0.076). However, no difference was found between Experiment 1 (85.8 ± 7.8 ms) and Experiment 3 (94.7 ± 7.6 ms; *P* = 1). On the other hand, in all the 3 experiments, the PSEs in baseline condition were larger than those in sound condition, *P*s < 0.01.

**Figure 2:**
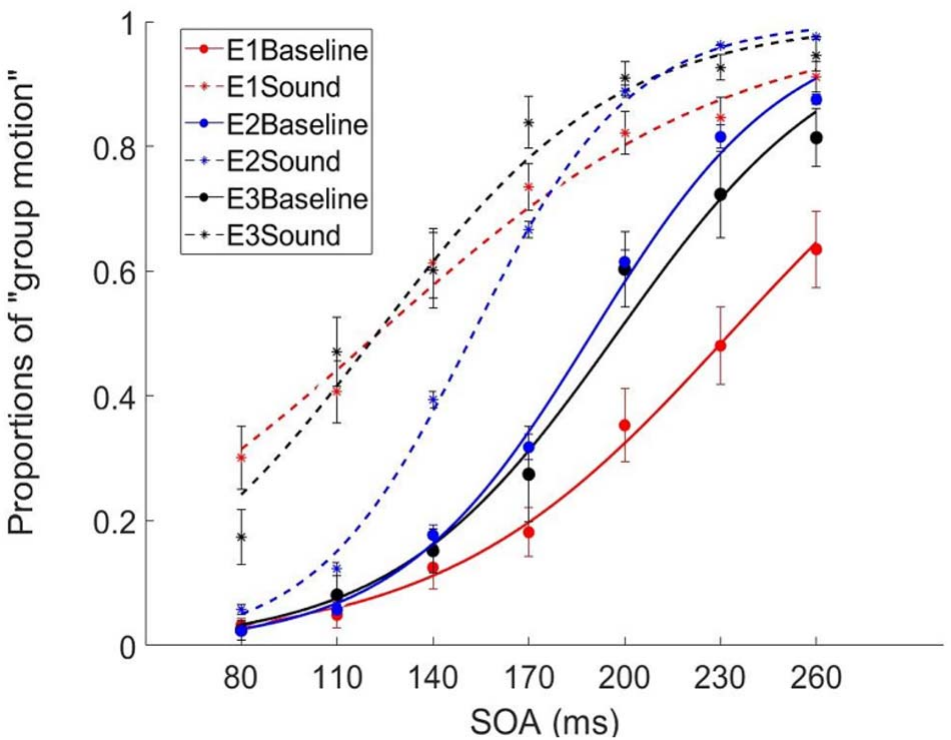
Proportions of the dominant percept of “group motion” as a function of different experimental conditions across 7 SOAs, parameterized with sound conditions. E1 (red lines): Experiment 1 used fully randomized trials; E2 (blue lines): Experiment 2 used blocked baseline and sound trials; E3 (black lines): Experiment 3 used blocked SOA conditions. “Baseline” (solid lines): visual Ternus stimuli were presented without tones. “Sound” (dashed lines): visual Ternus stimuli were presented synchronously with a pair of tones. The 7 SOAs ranged from 80 to 260 ms with a step size of 30 ms.

Therefore, the trial-by-trial randomized treatment (Experiment 1) magnified the crossmodal freezing effect (Fig. 2).

The detailed response proportions of “group motion” of the 3 experiments are shown in Supplementary Figures 2–4. In Experiment 1, the mean PSEs (±SE) for the “baseline” (no-sound) and “sound” conditions were 214.2 (±13.6) ms and 85.8 (±11.8). The PSE in the sound condition was significantly smaller than in baseline [*t*(16) = 6.629, *P* < 0.001]. To minimize intertrial effects due to the unexpected presentation of sounds and unexpected SOAs, in Experiment 2 we presented sound stimuli in blocks (absent vs. present) and randomly selected SOAs from the 7 levels (80–260 ms). The block orders were counterbalanced across participants using a Latin-square design. The PSEs (±SE) for the baseline and sound conditions were 160.3 (±2.6) ms and 120.2 (±0.8). The PSE in the sound condition was significantly smaller than in baseline [*t*(16) = 15.271, *P* < 0.001]. Furthermore, Experiment 3 was used to examine how the blockwise presentation of another factor (SOA) would shape the psychometric performance as well as the gaze pattern. The PSEs (±SE) for the baseline and sound conditions were 165.8 (±6.3) ms and 94.7 (±6.1) ms. The PSE in the sound condition was significantly smaller than the 1 in baseline [*t*(17) = 9.079, *P* < 0.001].

The JNDs were 31.4 (±3.3) and 38.9 (±6.2) for the baseline and sound conditions, respectively [*t*(17) = −1.417, *P* = 0.174]. The mean JNDs for Experiment 1, 2, and 3 were 56.5 (±5.1) ms, 29.1 (±5.2) ms, and 35.1 (±4.9) ms, respectively. The main effect of experiment was significant [*F*(2,48) = 7.943, *P* = 0.001, *η*^2^ = 0.249]. Bonferroni-corrected comparisons showed that the mean JND in Experiment 1 were larger than those in Experiment 2 (*P* = 0.001) and Experiment 3 (*P* = 0.012); however, no differences were found between the JNDs of Experiment 2 and Experiment 3 (*P* = 1). The interaction between experiment and condition was not significant [*F*(2,48) = 2.379, *P* = 0.103, *η*^2^ = 0.090]. Therefore, the randomized treatment of experimental trials (Experiment 1) produced larger JNDs, which made the discrimination more difficult.

**Figure 3:**
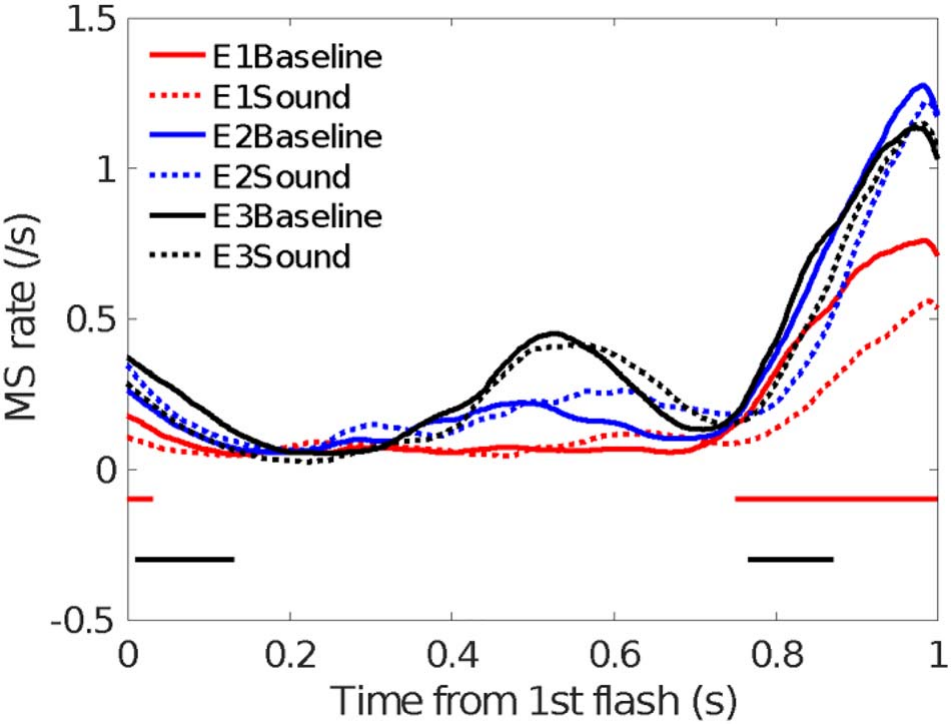
Microsaccades (MS) and their time courses in baseline and sound conditions. The horizontal colored lines indicate significant differences at *P* < 0.05 in the cluster analysis and correspond to the color code for each experiment.

When time-locked to the first visual frame, sound-induced OMI was significant in Experiment 1 (during the first visual frame presentation until 66 ms, and 738 ms after the first visual frame) and Experiment 3 (from 16 to 122 ms, and 770 to 874 ms after the first visual frame); however, no significant effect was found in Experiment 2 (*P* > 0.1) (Fig. 3). Importantly, the main effect of experiment was significant in terms of microsaccades. The mean MS rates were 0.161 (±0.046), 0.288 (±0.047), and 0.329 (±0.044) for Experiments 1–3, respectively. The MS rate was the smallest in Experiment 1 [F(2,48) = 3.739, *P* = 0.031, *η*^2^ = 0.135] (Experiment 1 vs. Experiment 3, *P* = 0.033; Experiment 1 vs. Experiment 2, *P* = 0.177; Experiment 2 vs. Experiment 3, *P* = 1).

This finding suggests that the fully randomized conditions lead to the largest MS inhibition effect, which obeys the inverse effectiveness principle consistent with the behavioral findings.

### Psychometric Findings (Average)

**Figure 4:**
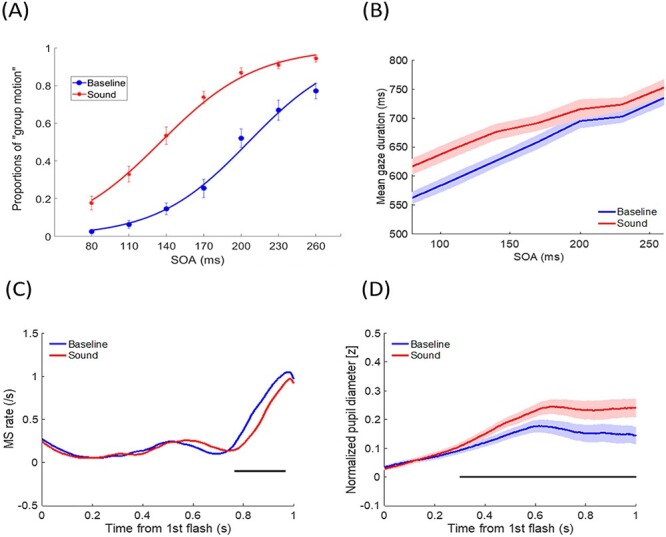
Psychometric results, gaze durations, microsaccades (MS) rate and normalized pupil dilations for Experiments 1–3 (pooled data). (*A*) Psychometric curves of observers judging the Ternus display as “group motion” across 7 SOAs, parameterized with sound conditions. (*B*) Mean gaze durations as a function of SOAs, parameterized with sound conditions. (*C*) MS rate change time-locked to the onset of the first Ternus frame, parameterized with sound conditions. (*D*) Mean pupil size change time-locked to the onset of the first Ternus frame, parameterized with sound conditions. The error bars in (*A*) and shaded areas in (*B*) and (*D*) represent the SE of mean across the participants. The black horizontal lines in (*C*) and (*D*) indicate significant differences at *P* < 0.05 (cluster analysis).

The averaged PSEs (± SE) for the baseline and sound conditions were 180.1 (±5.1) ms and 100.2 (±4.5) ms, respectively (data collapsed from Experiments 1–3). The PSE in the sound condition was significantly smaller than baseline [*F*(1,48) = 127.31, *P* < 0.001, *η*^2^ = 0.726], indicating a dominant percept of “group motion” during the Ternus display with synchronous sounds (Shi et al. 2010). The averaged JNDs (±SE) for the baseline and sound conditions were 37.9 (±3.0) ms and 42.6 (±3.7) ms, respectively. In general, the sounds did not affect the sensitivity of discriminating Ternus motion [*F*(1,48) = 1.865, *P* = 0.178, *η*^2^ = 0.037] (Fig. 4*A*), but significantly biased the percept to “group motion.”

### Gaze Duration

Overall, gaze duration was longer for the “sound” condition (688.8 ± 12.4 ms) than for “baseline” condition (652.6 ± 9.9 ms) [*F*(1,48) = 22.672, *P* < 0.001, *η*^2^ = 0.321]. The sound-prolonged gaze duration effect was more pronounced in the short than in long SOA conditions [*F*(6,288) = 4.898, *P* < 0.001, *η*^2^ = 0.093] (Fig. 4*B*).

### Oculomotor Inhibition

**Figure 5:**
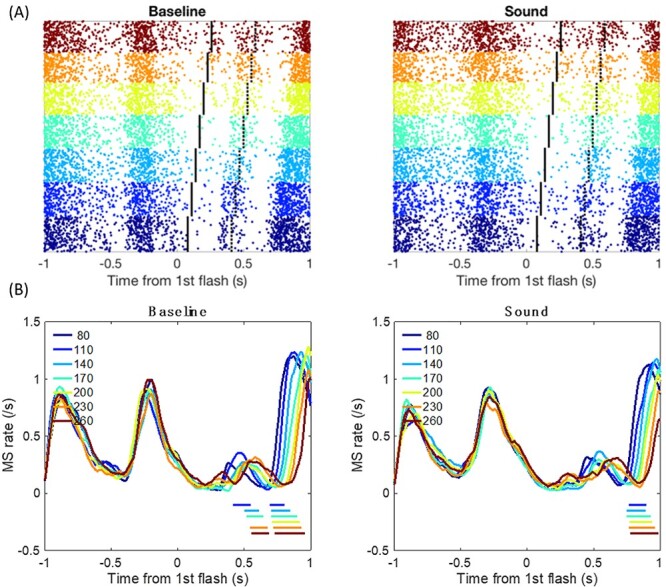
Microsaccade (MS) rates time-locked to the first flash onset in response to the Ternus display with different SOAs, separated by sound conditions. (*A*) The raster plots illustrate accumulated MS from all data. Each horizontal line represents 1 trial. Each dot represents a MS observed at the corresponding time point. Vertical black lines indicate the onset of the second flash. Vertical dotted lined indicate the onset of the question mark (for the participant to make a saccade to the peripheral question mark’s location). (*B*) MS rate change by time. The horizontal color lines indicate significant clusters (*P* < 0.05) between each color-coded SOA condition and the SOA = 80 condition.

Clear oculomotor fluctuation was found to correspond to the visual frames: early inhibition started around 400–550 ms after the first visual frame, in accordance with the SOAs (Fig. 5), where the MS rates were higher for longer SOAs in only the baseline condition and not in the sound condition. After 700 ms (the response phase), there was a delay of the OMI rebound, in which the MS rate was lower for longer SOA conditions in both the baseline and sound conditions. Most importantly, on average, that is, when collapsing all SOAs from Experiments 1–3, the MS rate was lower in the sound condition compared with the baseline condition 764–968 ms after the first visual frame, indicating that the MS rate was suppressed for longer in the sound condition (Fig. 4*C*). When segregating the analysis by different SOAs, the sound-induced OMI was constantly observed in most SOA conditions except for the longest 260-ms SOA condition (see Supplementary Figure 5).

**Figure 6:**
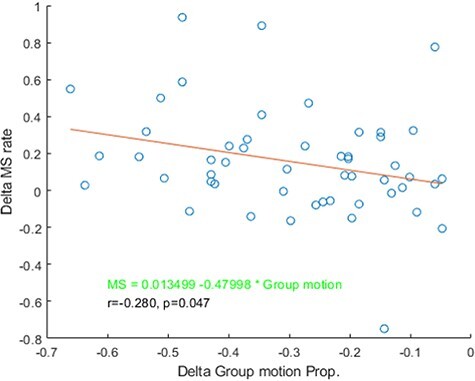
The delta microsaccade (MS) rate as a function of delta proportions reporting “Group motion.” Each dot represents data from each participant.

We calculated the correlation between the sound-induced OMI (500–1000 ms respective to the onset of the first frame) and subjective reports of “group motion” across all participants from Experiments 1–3. The difference in MS rate and “group motion” reports between the sound and baseline conditions represented delta MS rate and delta “group motion” report proportions, respectively. In the later time segment (500–1000 ms) locked to the onset of the first visual frame, we found a significant negative correlation between the 2 variables (*r* = −0.280, *P* < 0.05, Fig. 6). Further analysis showed that this correlation pattern was mainly attributed to Experiment 1 *(r* = −0.537, *P* = 0.026) and not Experiment 2 (*r* = −0.392, *P* = 0.133) or 3 (*r* = 0.245, *P* = 0.327). The result indicated that the participants who had stronger OMI tended to report “group motion” in the presence of concurrent sounds.

### Pupil Diameter Change

**Figure 7:**
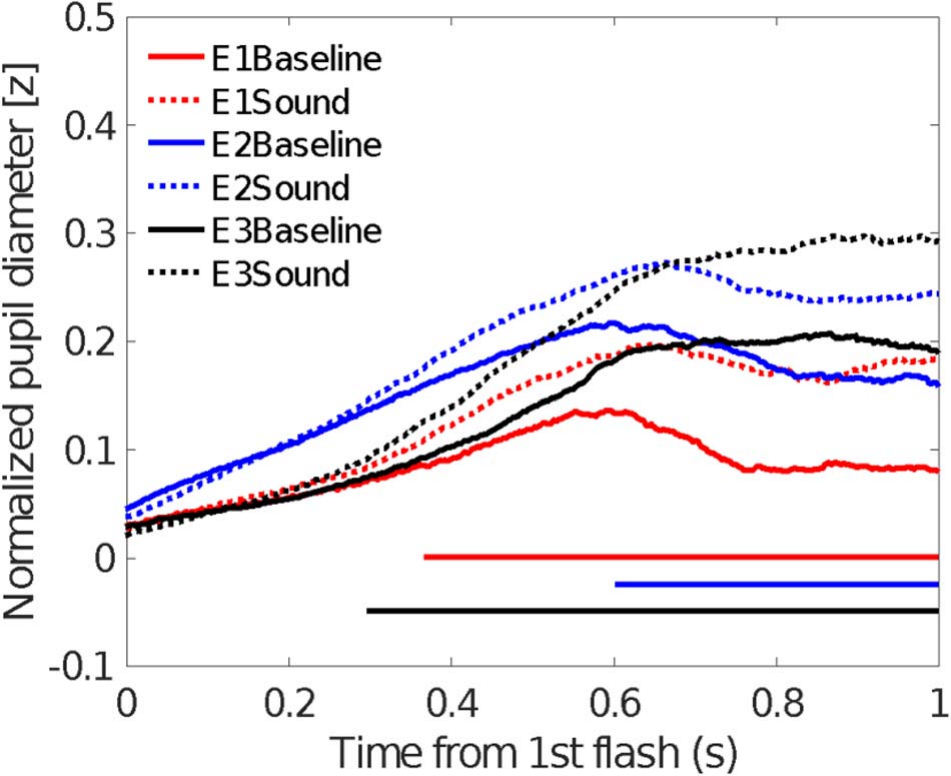
Mean pupil sizes in both baseline and sound conditions. The horizontal colored lines indicate significant differences at *P* < 0.05 in the cluster analysis and correspond to the color code for each experiment.

Pupil size increased more in the sound than in the baseline condition and a significant difference was observed 298 ms after the first visual frame (Fig. 4*D*, data combined for all SOAs and Experiments 1–3). This sound-induced PDR was consistently observed across almost all SOAs (except for 110 ms) and experiments at similar time points (see, Fig. 7 and Supplementary Figure 6).

For the 3 main Experiments, the potential confounding factor of involuntary blinks or gaze patterns in the baseline and sound conditions were ruled out. Blink rates remained identical for both conditions (Supplementary Figure 7). In addition, the gaze patterns indicated that participants followed the instructions and no apparent differences were observed between the baseline and sound conditions. This is shown in the heat map of gaze position (Supplementary Figure 8).

### Control Experiment (Experiment 4): Non-Ternus Visual Localization Task

#### Behavioral RT

**Figure 8:**
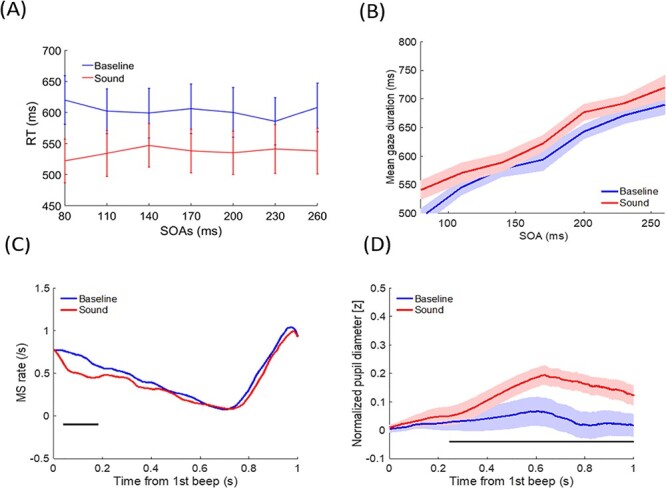
Results for the control experiment (visual localization) (*A*) RT as a function of SOAs, parameterized with sound conditions. (*B*) Mean gaze duration as a function of SOAs, parameterized with sound conditions. (*C*) MS rate change, time-locked to the onset of the first Ternus frame, parameterized with sound conditions. (*D*) Mean pupil size change time-locked to the onset of the first Ternus frame, parameterized with sound conditions. The error bars in (*A*) and shaded areas in (*B*) and (*D*) represent the SE of mean across the participants. The black horizontal lines in (*C*) and (*D*) indicate significant differences at *P* < 0.05 (cluster analysis).

Mean RT for locating targets were shorter in the sound (537 ± 36 ms) than in the baseline (no-sound) condition [603 ± 38 ms; *F*(1,102) = 49.769, *P* < 0.001, *η*^2^ = 0.745]. The main effect of SOA was not significant [*F*(6,102) = 0.448, *P* = 0.845, *η*^2^ = 0.026]. The interaction between sound condition and SOA was significant [*F*(6,102) = 3.109, *P* = 0.008, *η*^2^ = 0.155]: the effect size was relatively small in the SOA = 230 ms condition. The simple effects analysis did not show differences in RTs for any SOAs in either the sound or baseline conditions (*P* > 0.1). Therefore, only a general sound facilitation/alerting effect for localization was observed (Fig. 8*A*).

#### Gaze Duration

The mean gaze durations were larger in the sound (630.4 ± 13.2 ms) than in the baseline condition [602.2 ± 12.8 ms; *F*(1,17) = 29.676, *P* < 0.001, *η*^2^ = 0.636], and increased as a function of SOA [510.2 (±17.3) ms, 543.5 (±19.9) ms, 579.6 (±19.0) ms, 598.4 (±19.7) ms, 650.9 (±15.1) ms, 668.7 (±18.8) ms, and 693.3 (±20.2) ms for SOAs = 80–260 ms in 30 ms increments *F*(6,102) = 112.006, *P* < 0.001, *η*^2^ = 0.868]. The interaction between sound condition and SOA was not significant [*F*(6,102) = 0.653, *P* = 0.688, *η*^2^ = 0.037] (Fig. 8*B*). In contrast to the main experiment results, sound prolonged gaze duration in general, regardless of SOA.

#### Oculomotor Inhibition

OMI was mostly observed at the later time range (~700 ms after the first beep), and corresponded to the SOA conditions, independent of the sound conditions (Supplementary Figure 9). Most importantly, sound-induced OMI was observed at a very early time range of 40–182 ms after the first beep (Fig. 8*C*). Moreover, sound-induced OMI was most frequently observed in the 200 ms SOA condition (32–162 ms) but not in the other SOA conditions (Supplementary Figure 10). The overall pattern of this result differed considerably from what was observed in the Ternus display experiments.

#### Pupil Diameter Change

Similar to the observation during the Ternus display experiments, pupil size increased more in the sound than in the baseline condition and the deviation started 244 ms after the first beep (Fig. 8*D*). This sound-induced PDR was observed in all SOA conditions: starting at 398 ms for 80 ms SOA, 424 ms for 110 ms SOA, 382 ms for 140 ms SOA, 352 ms for 170 ms SOA, 672 ms for 200 ms SOA, 402 ms for 230 ms SOA, and 396 ms for 260 ms SOA.

### Comparison Between the Ternus Apparent Motion Tasks (Experiments 1–3) and the Visual Localization Task (Experiment 4)

To investigate whether the sound-induced eye metric characteristics were specific to Ternus apparent motion judgments (which requires audiovisual integration), we conducted between-experiment comparisons of OMI and pupil diameter change. The MS rate and pupil size change data were segmented into the 4 following time ranges: 0–250 ms (S1), 250–500 ms (S2), 500–750 ms (S3), and 750–1000 ms (S4), and subjected to a mixed-model ANOVA, with sound condition (sound, baseline) and time segment (S1–S4) as the within-subject factors and task demand (Ternus task, visual localization task) as the between-subject factor.

#### Oculomotor Inhibition

The mean MS rates for both the Ternus and localization tasks were 0.260 (±0.028) and 0.433 (±0.047) [*F*(1,67) = 9.985, *P* = 0.002, *η*^2^ = 0.130]. The two-way interaction between task and segment was significant [*F*(3,201) = 12.737, *P* < 0.001, *η*^2^ = 0.160]. The simple effects analysis indicated that for S1 and S2, the MS rates were lower in the Ternus task than in the localization task (both *P* < 0.001), but no differences were found for S3 (*P* = 0.535) or S4 (*P* = 0.953).

The three-way interaction between task, sound conditions, and segment was significant [*F*(3,201) = 3.235, *P* = 0.023, *η*^2^ = 0.046]. In both sound and baseline conditions, across S1 and S2, the MS rates were lower in the Ternus task than in the localization task (*Ps* < 0.001). The two-way interaction between sound condition and segment was significant [*F*(3,201) = 5.099, *P* = 0.002, *η*^2^ = 0.071]. The simple effects analysis indicated that for S1 and S4, the MS rates in the sound conditions were lower than those in the baseline condition (*Ps* < 0.01). However, for S2 and S3, MS rates were not significantly different (all *P* > 0.1). For the Ternus task, the interaction of sound condition and segment was significant [*F*(3,150) = 12.871, *P* < 0.001. *η2* = 0.205]. The MS rate was larger in the sound (0.213 ± 0.045) than in the baseline condition (0.169 ± 0.038) for S3 (*P* = 0.028), but smaller in the sound (0.535 ± 0.061) than in the baseline condition (0.690 ± 0.069) for S4 (*P* < 0.001). This indicated that the OMI was followed by the rebound of MS. In contrast, for the localization task, the interaction between sound condition and segment was not significant [*F*(3,51) = 1.2, *P* = 0.319, *η*^2^ = 0.066]. These findings suggest that the freezing effect in audiovisual integration was driven by OMI, with the critical time course of inhibition and rebound occurring during S3 and S4 of the sound condition.

#### Pupil Diameter Change

The data were segmented into 4 time ranges as in the OMI analysis. The normalized pupil diameters for both baseline and sound conditions were 0.078 (±0.017) and 0.140 (±0.016) [*F*(1,67) = 73.617, *P* < 0.001, *η*^2^ = 0.524]. The three-way interaction between task, sound condition, and segment was not significant [*F*(3,201) = 2.182, *P* = 0.091, *η2* = 0.032]. The main effect of segments was significant [*F*(3,201) = 17.519, *P* < 0.001, *η*^2^ = 0.207]. Bonferroni-corrected comparisons identified the smallest diameter in S1 (*P* < 0.01), and an increased diameter in S2 (with S2 vs. S1, *P* < 0.001; S2 vs. S3, *P* < 0.001); however, no difference was found between S2 and S4 (*P* = 0.313) or between S3 and S4 (*P* = 1).

The mean pupil diameters for the Ternus and localization tasks were 0.144 (±0.016) and 0.074 (±0.027) [*F*(1,67) = 4.949, *P* = 0.029, *η*^2^ = 0.069]. The two-way interaction between task and segment was significant [*F*(1,67) = 6.755, *P* = 0.011, *η*^2^ = 0.092]. Further analysis indicated that the larger pupil size in the Ternus than in the localization task was more prominent during the S2 period (*P* = 0.046) than during the other segments.

The two-way interaction between sound condition and segment was significant [*F*(3,201) = 48.337, *P* < 0.001, *η2* = 0.419]. The simple effects analysis indicated that across S2-S4, the diameters of sound-present conditions were larger than those of the baseline condition (*P* < 0.001). However, S1 showed no difference in pupil diameter (*P* = 0.089). The overall results indicated that pupil size increased by time, regardless of task.

### OMI Locked to the Onset of the Second Visual Ternus

As visual flashes heavily influenced MS, 1 might argue that the result of MS rate time-locked to the first Ternus visual flash would be influenced by the second flash, which might interact with the effect of the sound. To clarify the issue, we conducted further analysis of OMI, in which the MS rate data were time-locked to the second Ternus flash (see Supplementary Figures 11–14). Results showed that the MS rate was higher in the sound condition compared with the baseline condition during 410–568 ms and lower in the sound than the baseline condition during 620–796 ms after the second visual frame, indicating a clear effect of sound modulation on the MS rate. Results of between-experiments comparison showed that the mean MS rates were 0.275 (±0.056), 0.467 (±0.058), and 0.474 (±0.055) for Experiments 1–3, respectively. The main effect of experiments (Experiments 1–3) was significant, *F*(2,48) = 4.026, *P* = 0.024, *η*^2^ = 0.144. The MS was the smallest in Experiment 1 (Experiment 1.vs. Experiment 2, *P* = 0.066; Experiment 1 vs. Experiment 3, *P* = 0.043). Sound-induced OMI and this effect was mainly observed at the relatively late stage (S3, 500–750 ms) before it attenuated in the final temporal segment (S4, 750–1000 ms). For the control experiment, when time-locked to the onset of the second beep, no significant sound-induced OMI was found in the control experiment (see Supplementary Figures 15–17). The cross-experiments analysis indicated the sound-induced OMI effect in Ternus task (Experiments 1–3) was larger than 1 in localization task (Experiment 4) upon the onset of second visual Ternus (0–250 ms), showing OMI modulation is task-specific.

## Discussion

### Crossmodal Perceptual Grouping by Eyes and Ears: Attention and Sensory Reliability-Related Microsaccade Changes

The Ternus display served as an excellent paradigm to study the neural correlates of nonretinotopic, relative motion perception. This paradigm is demonstrated to be a versatile tool for the study of nonretinotopic processing without eye movements (saccades), since the visual frame elements are located within the central fovea area for observation (Boi et al. 2011; Pooresmaeili et al. 2012; Thunell et al. 2016). In the present study, the synchronous sound inputs triggered more reports of “group motion” in the Ternus display, replicating previous behavioral results that used the same paradigm (Shi et al. 2010; Chen et al. 2018).

To the best of our knowledge, the temporal dynamics of perceptual grouping in audiovisual integration have not been studied empirically using an eye tracking approach (in particular within short time ranges of 1 s), except for a few studies on spatial orienting (Rolfs et al. 2005; Wang et al. 2017). Temporal ventriloquism is an effective paradigm for studying the crossmodal perceptual grouping effect (Vroomen and de Gelder 2000; Freeman and Driver 2008; Chen et al. 2010; Shi et al. 2010; Chen and Vroomen 2013). In temporal ventriloquism, paired beeps or salient sounds (grouped by similar pitch) segregate the corresponding and concurrent visual events. Consequently, the observer easily identifies the visual targets and thus, biases visual motion perception (Freeman and Driver 2008; Shi et al. 2010; Chen et al. 2011; Roseboom et al. 2013; Chen *et al*. 2018).

Previous studies have revealed that saccading to a relevant stimulus can be an overt correlate of the allocation of spatial attention, whereas precisely timed gaze stabilization can be an overt correlate of the allocation of temporal attention (Denison et al. 2019). The present study contributes to our understanding of the MS mechanism during a covert attention paradigm. We did not overtly direct attention to the spatial directions of the target, as shown in most previous studies (Hafed and Krauzlis 2010, 2012; Wang et al. 2017). In our case, upon receiving the auditory input, with the attentional demands on the visual input, observers employed a nonretinotopic binding of audiovisual events to establish distinctiveness of visual objects (with accompanying sounds) across space and time (Otto et al. 2010), which results in inhibited microsaccades (Siegenthaler et al. 2014; Krzysztof et al. 2018). Typically, this process resulted in a delayed time course of OMI (Fig. 2*C*, Supplementary Figure 3 and 4), in response to either attentional selection under a demanding task or the subjective prolonging of visual event durations with concurrent auditory inputs (Wearden et al. 1998; Shi et al. 2010). The OMI last longer and we observed it existed until S3 (500–750 ms) after the onset of the second visual Ternus frame, before its rebound in S4 (750–1000 ms). The OMI pattern is task-specific since we found more deep modulation in Ternus task than the 1 in control (“localization”) task. As we have shown, the change magnitude of the microsaccade rates is typically low. Indeed, the low-change signals (in presence of sounds) were generated in advance, and could be used as an optimal (discrete) temporal sampling strategy (Martinez-Conde et al. 2009; Rolfs 2009), to resolve the perceptual ambiguities as typified in the visual Ternus display. Microsaccades (“oculomotor freezing”) observed in the present study, go beyond vision and indicate a crossmodal coupling between oculomotor action and temporal attending among different sensory modalities (Badde et al. 2020). In this means, microsaccades, though rare and with low changes in amplitudes, still provide highly sufficient information about the temporal attending and its temporal dynamics during brief audiovisual integration (Pastukhov and Braun 2010).

We found a coherent link between the behavioral effect-size and the MS rate across different experimental conditions. Specifically, in Experiment 1 we varied the presence of sound and the SOA between the 2 visual Ternus frames, in a fully randomized manner. This arrangement imposed the largest uncertainty (as well as a high attentional demand) and greatly reduced the expectation of the trial properties. Accordingly, observers showed the largest bias in perceptual classification of Ternus motion (with greatly reduced PSE) as well as the sensitive readout of eye metrics (i.e., greatest inhibition of microsaccades). By recording microsaccades, we demonstrated that during crossmodal integration, the uncertainty of the stimuli presentation affected the OMI as well, resembling the “inverse effectiveness” as shown in pervious findings (Holmes 2009; Hou et al. 2019).

### Microsaccades as a Temporal Trigger

Microsaccades are generated when fixation-related activity at the rostral center of the superior-colliculus map spreads to neighboring locations due to local excitation (Engbert and Kliegl 2003; Rolfs et al. 2008a; Amit et al. 2019). In our case, attention on auditory events could serve as a temporal trigger, shift the balance of oculomotor (spontaneous) maps, and favor or even consolidate the nonretinotopic channel, which binds the audiovisual events and produces the dominant percept of “group motion” (Park et al. 2019). This suggests that, although rare, the occurrence of MS is a sampling strategy (Mergenthaler and Engbert 2007; Martinez-Conde et al. 2009). This has been shown to be robust in a previous unimodal study, where MS are persistent until a perceptual decision is made (Pastukhov and Braun 2010; Widmann et al. 2014; Loughnane et al. 2018).

### MS Rate Prevails Over Pupil Dilation in Audiovisual Integration in Brief Temporal Scale

In our study, the crossmodal integration effect was not contributed to by saliency detection that featured with the PDR, but rather by OMI. The genuine crossmodal freezing effect was specific to the Ternus task, where audiovisual integration is required. In contrast, the control experiment (Experiment 4) with visual localization did not generate a compatible gaze duration pattern as observed in the Ternus tasks (Experiments 1–3). Sound beeps triggered similar pupil dilations in both the Ternus tasks and localization task. A PDR was observed in the presence of sounds across all tasks, and pupil sizes increased more in the sound condition than in the baseline condition. OMI is a sensitive index for the discrimination of task-specific processing. With audiovisual integration, MS rates were lower in the Ternus task than in the localization task and the MS suppression lasted longer.

Our finding that sounds induce stronger PDR is consistent with previous findings (Wang et al. 2014; Liao et al. 2016a; Liao et al. 2016b), which likely relates to the superior colliculus (Wang et al. 2012) as the neural substrate for audiovisual integration. Given the very brief duration of the sounds, the changes in pupil size may not have been sensitive enough to indicate the temporal dynamics of crossmodal integration.

In sum, using the paradigm of visual Ternus display and eye movement metrics, we identified coherent behavioral and neuropsychological evidence for the temporal dynamics (within 1 s) during crossmodal integration: concurrent inputs of beeps “freeze” bi-stable visual apparent motion percepts to be one-way dominant, with characteristic sound-induced OMI.

## Notes

*Conflict of interest:* None declared.

## Funding

Project Crossmodal Learning of National Natural Science Foundation of China (NSFC) (NSFC 61527804, NSFC 61621136008, NSFC 31861133012); the Research Fund from Brain Lab, Tomorrow Advancing Life Education Group, China.

## Supplementary Material

Microsaccade_and_crossmodal_freezing_effect_supplement_tgaa072
