# Supplementary material for: Microsaccadic Eye Movements but not Pupillary Dilation Response Characterizes the Crossmodal Freezing Effect
Source: Cereb Cortex Commun. 2020 Sep 30;1(1):tgaa072. doi: 10.1093/texcom/tgaa072 (PMC8153075; doi:10.1093/texcom/tgaa072)
Supplement: Microsaccade_and_crossmodal_freezing_effect_supplement_tgaa072 [file microsaccade_and_crossmodal_freezing_effect_supplement_tgaa072.docx]

*Supplement Materials:*

**Microsaccadic eye movement but not pupillary dilation response characterizes the crossmodal freezing effect**

Lihan Chen^1,2,3*^, Hsin-I Liao^4*^

1 School of Psychological and Cognitive Sciences, Peking University, Beijing, 100871, China.

2 Beijing Key Laboratory of Behavior and Mental Health, Peking University, Beijing, China

3 Key Laboratory of Machine Perception (Ministry of Education), Peking University, Beijing, China
4 NTT Communication Science Laboratories, NTT Corporation, Atsugi, Kanagawa, Japan.

*Correspondence Email: CLH@pku.edu.cn, hsini.liao.pb@hco.ntt.co.jp

**Microsaccadic amplitude and peak velocity (for main experiments)**


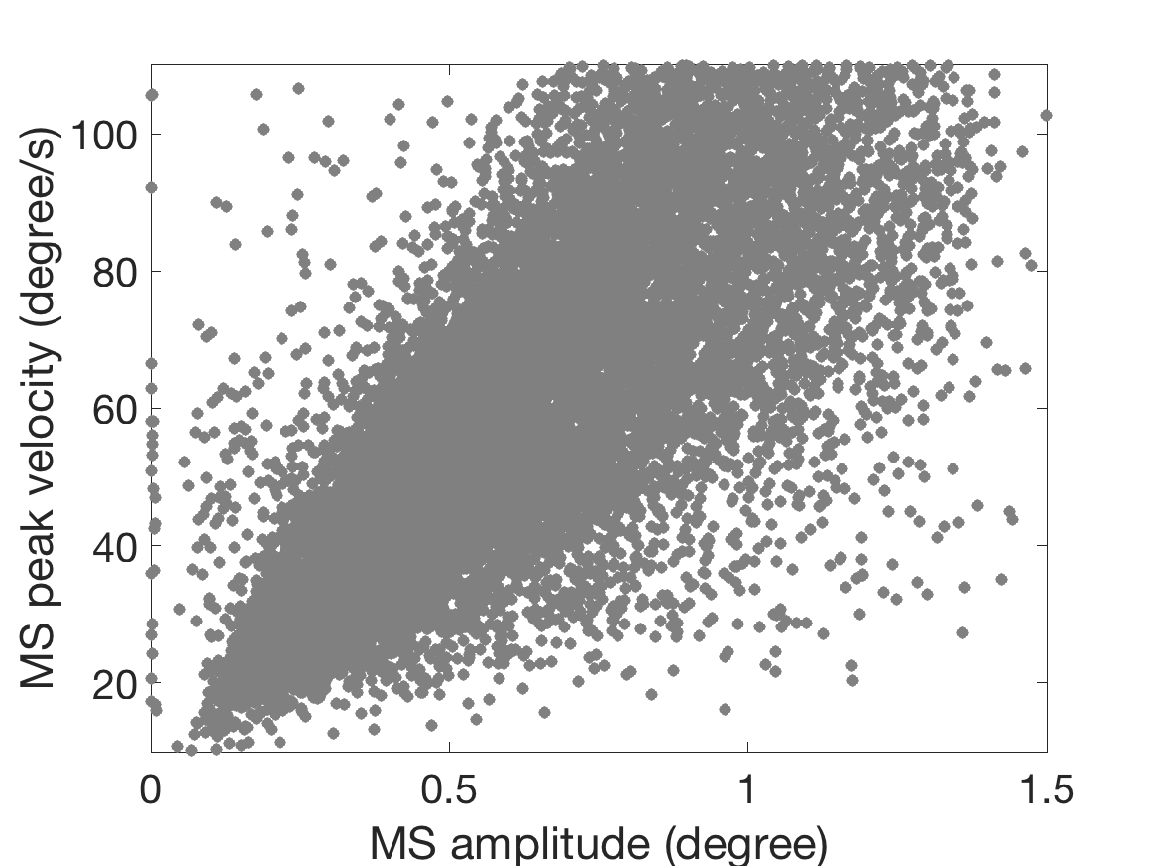


**Figure S1.** The correlation between the microsaccadic amplitude and peak velocity of all the detected microsaccades in the main three experiments.

**The mean proportions of reporting ‘Group motion’**


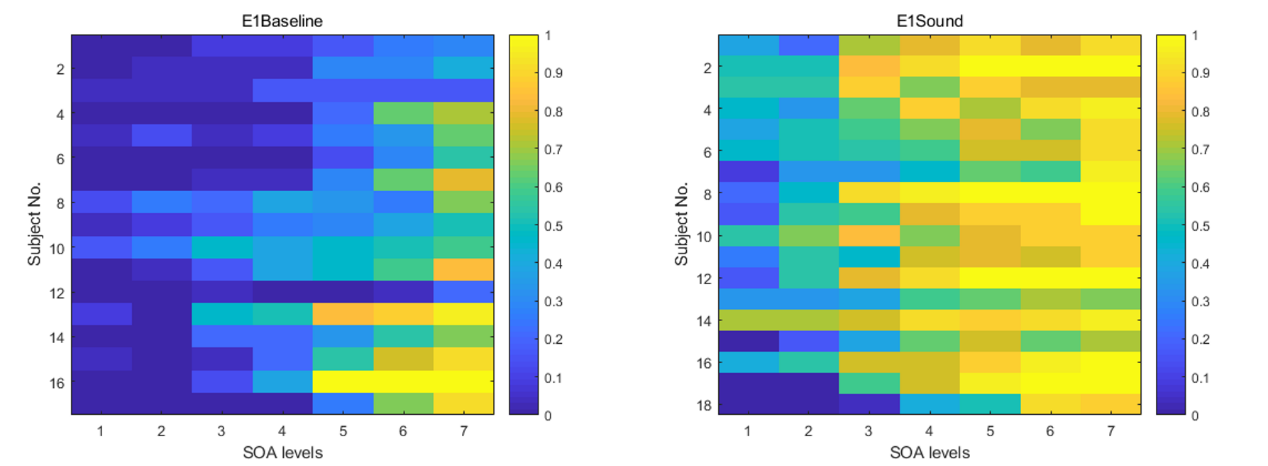


**Figure S2.**  The mean proportions of reporting ‘Group motion’ for each participant across seven SOA levels in both baseline and sound conditions in Experiment 1.


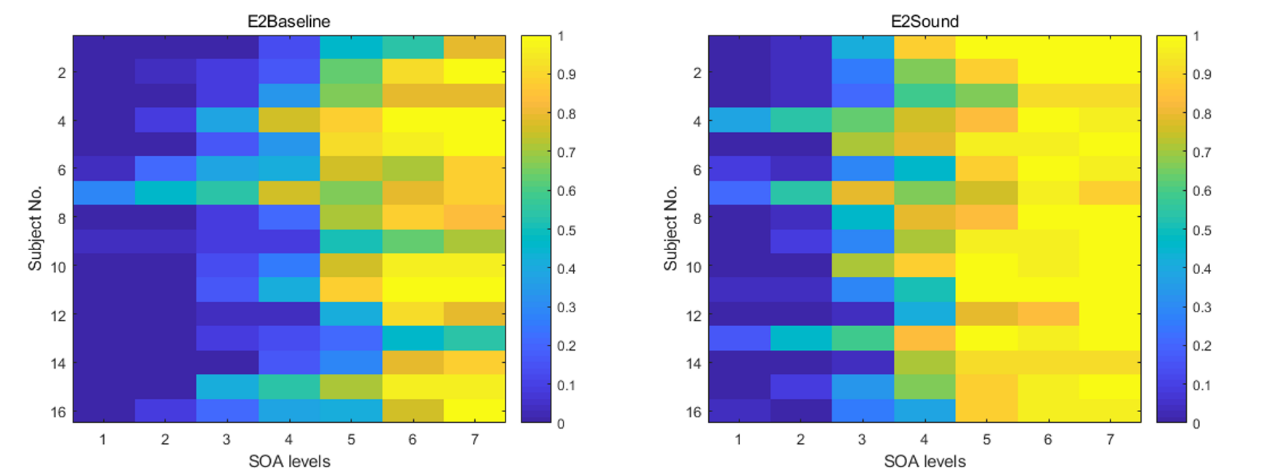


**Figure S3.**  The mean proportions of reporting ‘Group motion’ for each participant across seven SOA levels in both baseline and sound conditions in Experiment 2.


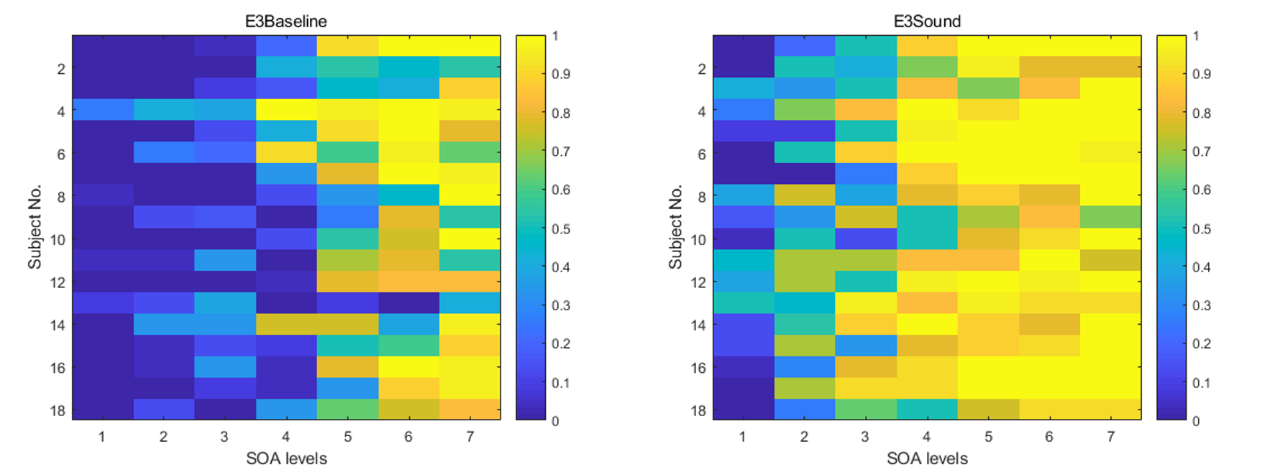


**Figure S4.**  The mean proportions of reporting ‘Group motion’ for each participant across seven SOA levels in both baseline and sound conditions in Experiment 3.

**Oculomotor inhibition in main experiments**


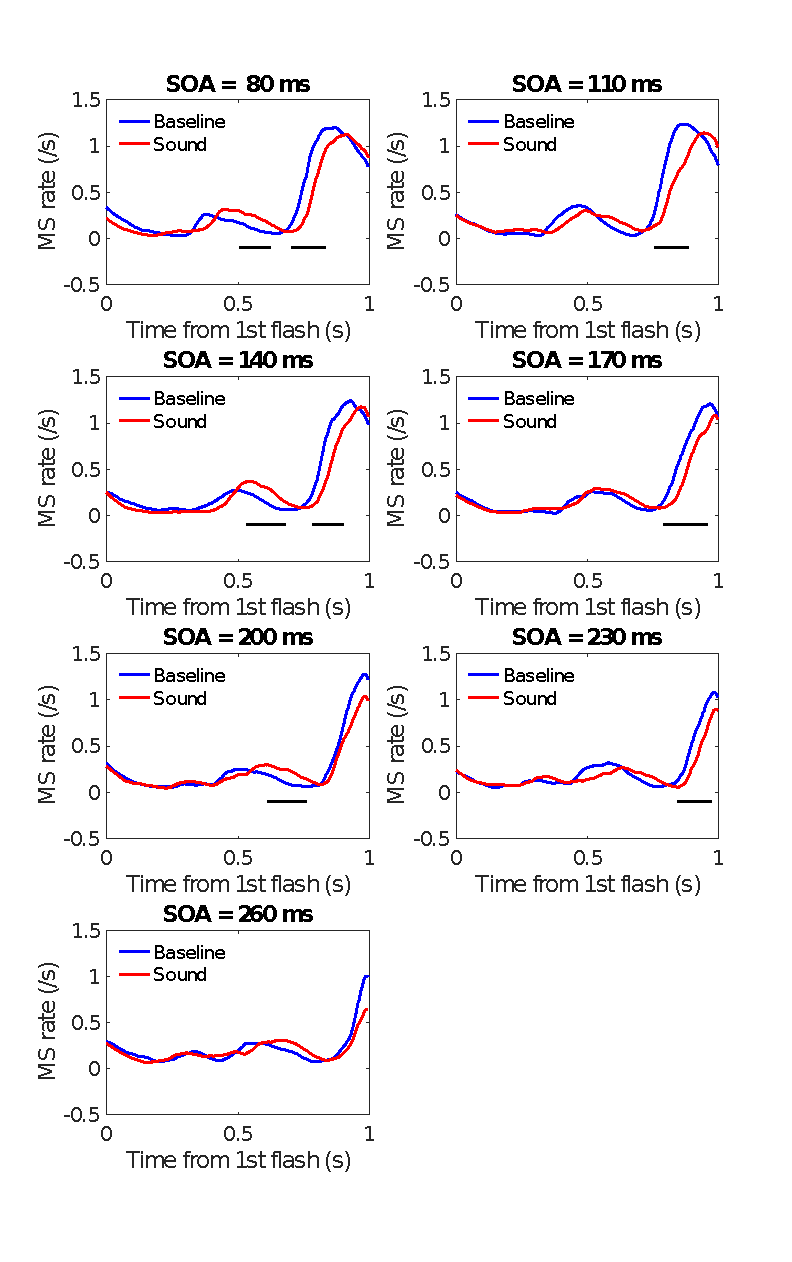


**Figure S5.** MS results for main experiments. MS rate as a function of the time from the first visual frame in both baseline (blue line) and sound (red line) conditions. The black horizontal lines indicate significant differences at the *p* < 0.05 level (cluster analysis).

**Pupil diameter change**

Sound-induced pupil dilation response was observed almost in all SOAs and experiments. For SOAs, the significance between the sound and baseline was ranged as the following: 302 ms for 80-ms SOA, 430 ms for 140-ms SOA, 440 ms for 170-ms SOA, 158 ms for 200-ms SOA, 388 ms for 230-ms SOA, and 388 ms for 260-ms SOA conditions. For experiments, the difference started from 366 ms, 602 ms, and 296 ms after the first visual frame in Experiments 1, 2, and 3, respectively.


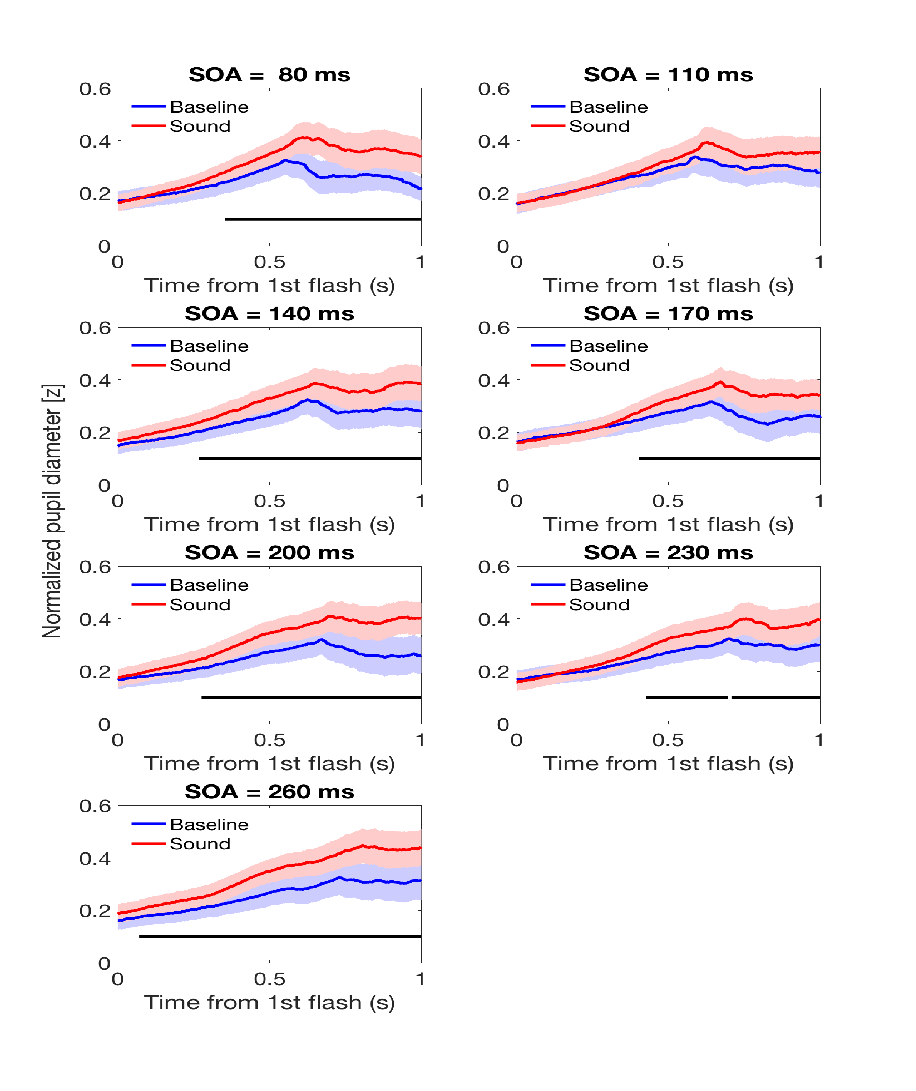


**Figure S6.** Mean pupil sizes in both baseline and sound conditions, separated by SOA conditions. The horizontal black lines indicate significant differences at the *p* < 0.05 level by cluster analysis.

**Blink control:**

For all three main experiments (E1-3), the potential confounding factors arising from involuntary blinks in either baseline or sound conditions were ruled out. The blink rates did not significantly differ from each other between the baseline and sound conditions


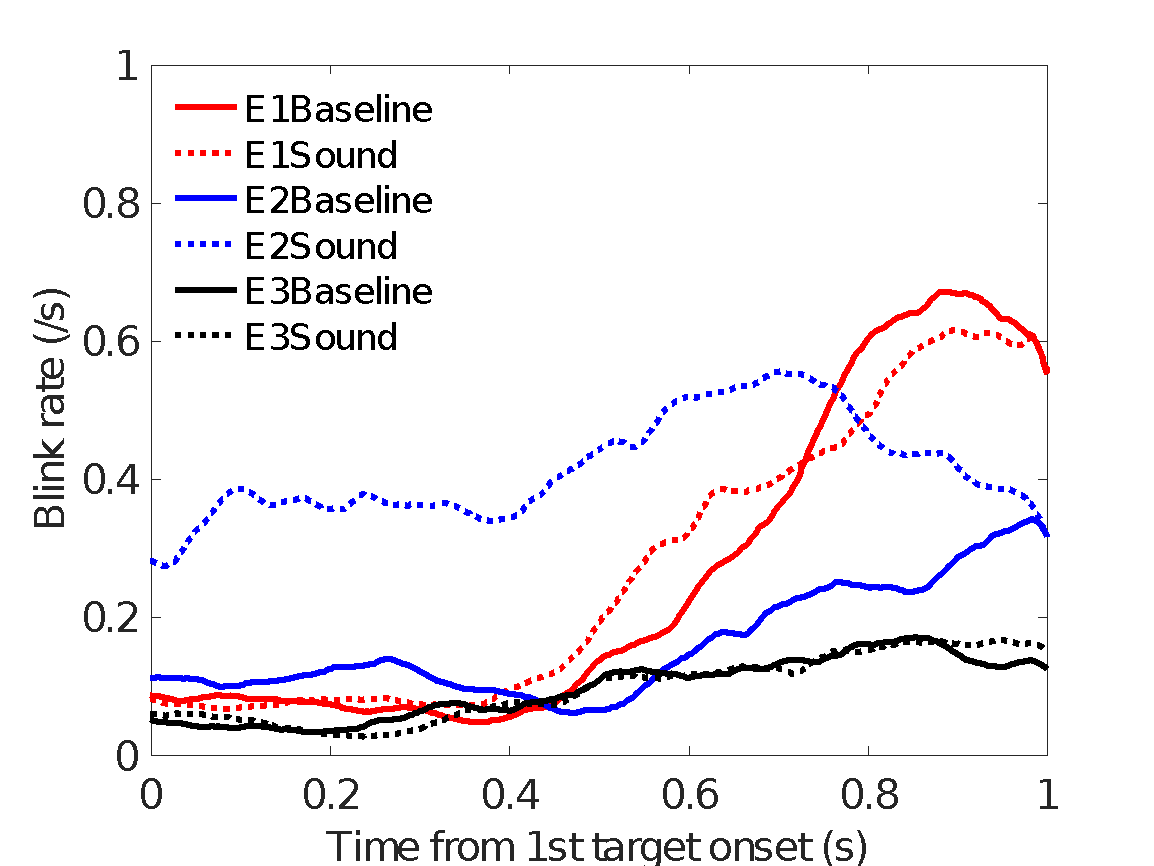


**Figure S7.** Blink rate as a function of time (0-1 sec), when the time unfolds across the onset of the first visual frame. No significant differences were found between baseline and sound conditions in any of the experiments.

**Heat map of gaze position**

The gaze positions were examined across different key events during experiments. The obtained gaze position patterns indicated that participants followed the instructions very well, by gazing at the centre at the start of the trials (Figure S8A), or by moving their gazes to left or right in response to task demands (Figure S8B).


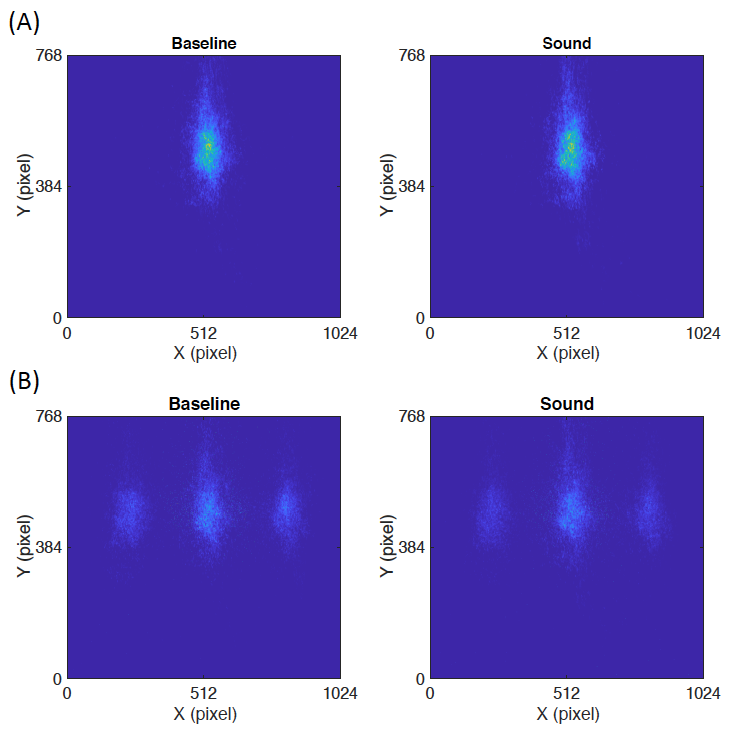


**Figure S8. Heat map of gaze position for E1-E3: (A)** Time window: 0-0.5 s, Participants focused at the centre, independent of the sound conditions. **(B)** Time window: 0.5-1 s, participants moved their gaze to the left or right in response to task demand, independent of sound conditions. The screen resolution (pixels) was 1024 (width) x 768 (height), with (512 and 384) as the centre point of the screen.

**Oculomotor inhibition in control experiment**

**Figure S9. MS rates time-locked to the 1^st^ beep in response to the visual localisation task with different SOAs, separated by sound conditions**. (A) Raster plots illustrate accumulated MS from all data. Each horizontal line represents one trial. Each dot represents a MS observed at the corresponding time point. Vertical black lines indicate the onset of the 2^nd^ beep. Vertical dotted lines indicate the onset of the question mark (for the participant to make a saccade to the peripheral question mark’s location). (B) MS rate change by time. The horizontal colour lines indicate significant clusters (p < 0.05) between each color-coded SOA condition and the SOA = 80 condition.


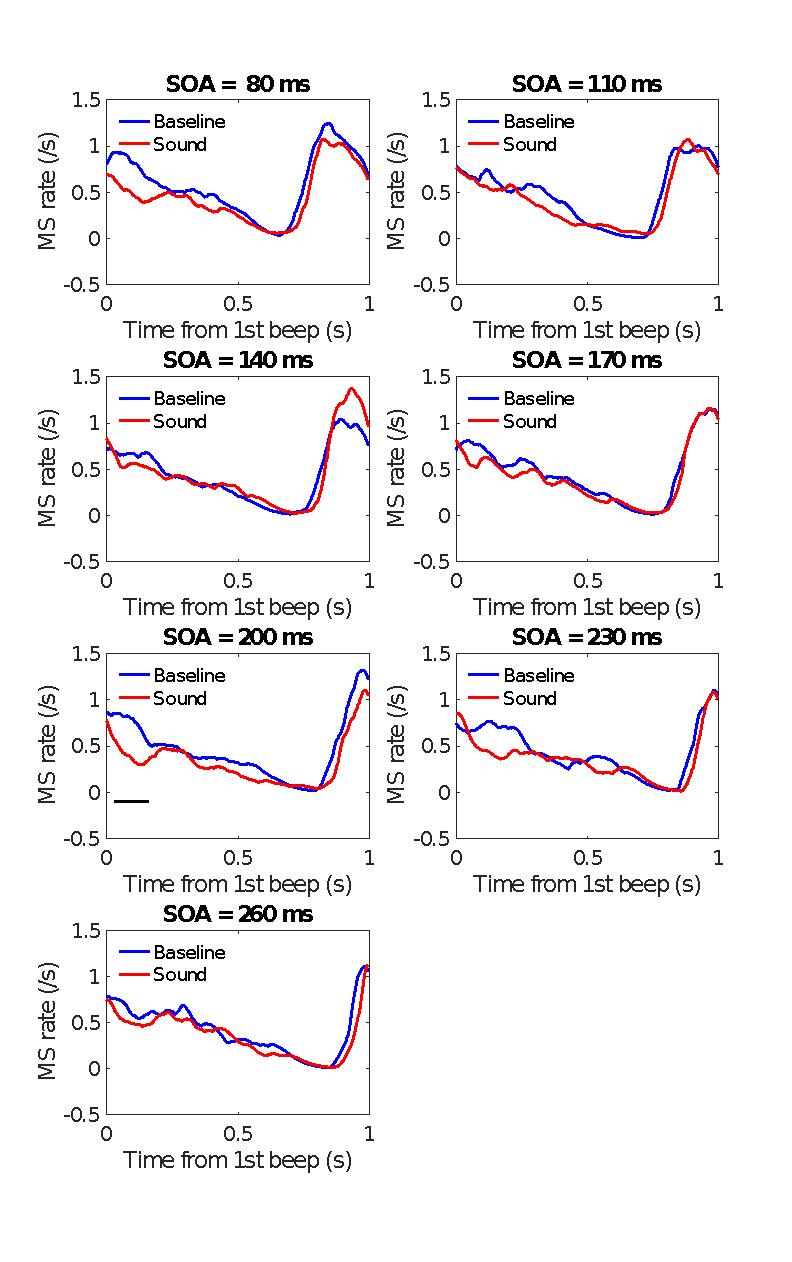


**Figure S10.** MS rate in response to the visual localisation task as a function of the time from the first beep in both baseline (blue line) and sound (red line) conditions. The black horizontal lines indicate significant clusters (p < 0.05) between baseline and sound conditions.

***Oculomotor inhibition locked on the onset of the second visual Ternus***

**Microsaccades locked on the 2nd visual Ternus frame (Experiments 1-3)**

When the OMI was locked on the onset of the second visual Ternus, the mean MS rates were 0.275 (±0.056), 0.467 (±0.058) and 0.474 (±0.055) for Experiments 1-3, respectively. The main effect of experiments was significant, *F*(2,48)=4.026, *p*=0.024, *η*^2^ = 0.144. The MS was the smallest in Experiment 1 (Exp 1.vs Exp.2, p=0.066; Exp.1 vs Exp.3, p=0.043). The mean MSs for baseline and sound conditions were 0.414 (±0.034) and 0.397 (±0.033) respectively [ *F*(1,48)=1.187, *p*=0.281, *η*^2^ = 0.024]. We separate the 1 second range (time locked to the onset of the 2nd visual frame) into four segments: 0-250 ms (S1), 250 ms -500 ms (S2), 500 ms- 750 ms(S3) and 750 ms-1000 ms(S4) and examined how the MSs evolved over time. The mean MSs for the four segments were 0.084 (±0.018), 0.216 (±0.051), 0.310 (±0.035), 1.012 (±0.079) for S1-S4, *F*(3,144)=83.72, *p*<0.001, *η*^2^ = 0.636. Generally, the MS become larger across the four segments. The interaction between segments and experiments was significant [*F*(6,144)=2.211, *p*=0.045, *η*^2^ = 0.084].The interaction between segments and conditions (baseline vs. sound) was also significant [*F*(3,144)=3.912, *p*=0.010, *η*^2^ = 0.075] .

Further simple effect analysis indicated that there was no statistical differences between S2 and S3 for MSs, the MS was lowest in S1 and largest in S4. For the S4, the MS in Experiment 2 (1.238 ±0.141) was larger than the MS in Experiment 1 (0.735 ±0.137), *p*=0.041.The inhibition of MS (OMI) was mainly observed in S3, in which the MS in sound condition was 0.254 (±0.033) while MS in sound condition was 0.365 (±0.044), *p*=0.003. In the sound conditions, except for the cohort S2 and S3 (p=1), the other cohorts (S1-S4) were significant in MS differences. The MSs increased across four segments in baseline (no sound) condition. Therefore, sound induced OMI and this effect was mainly observed at the relatively late stage (S3) before it attenuated in the final temporal segment (S4).


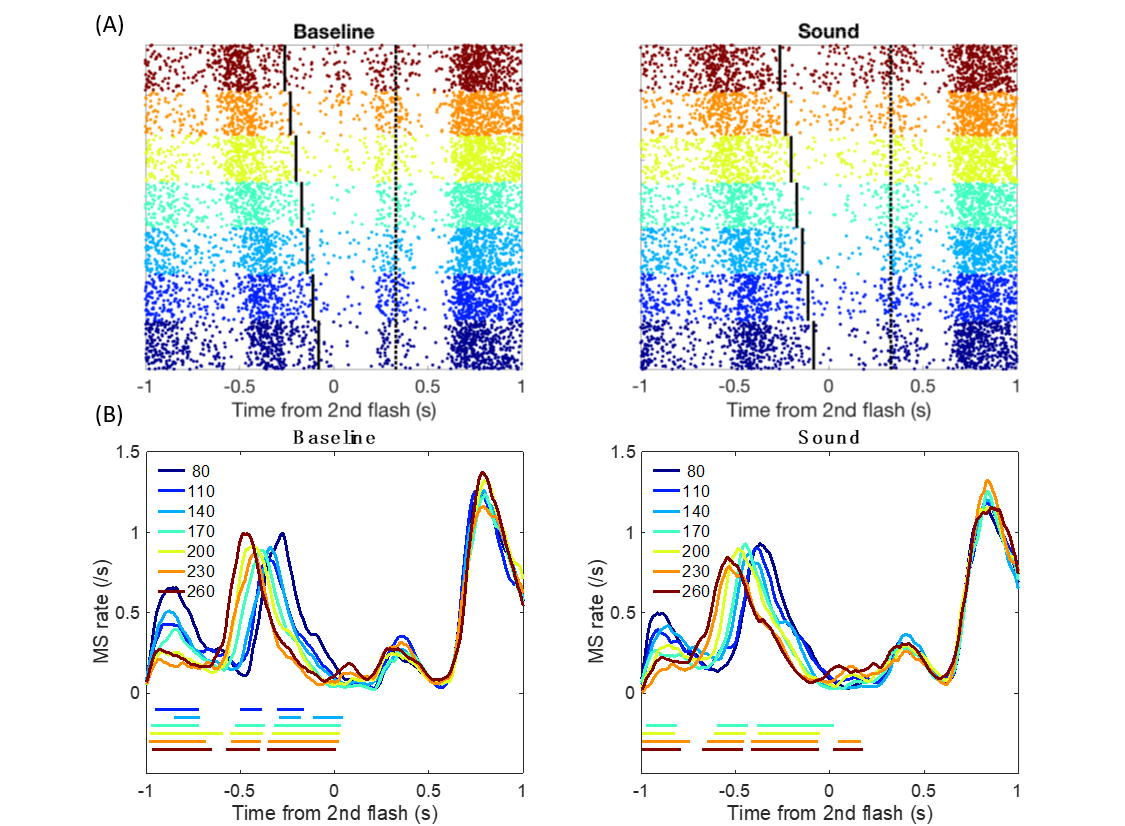


**Figure S11** **Microsaccade eye movement (MS) rates in response to the Ternus display with different SOAs, time-locked to 2^nd^ flash onset, separated by sound conditions.** (A) The raster plots illustrate accumulated MS from all data. Each horizontal line represents one trial. Each dot represents a MS observed at the corresponding time point. Vertical black lines indicate the onset of 1^st^ flash. Vertical dotted lines indicate the onset of the question mark (for the participant to make a saccade to the peripheral question mark’s location). (B) MS rate change by time. The horizontal color lines indicate significant clusters (*p* < 0.05) between each color-coded SOA condition and the SOA = 80 condition.


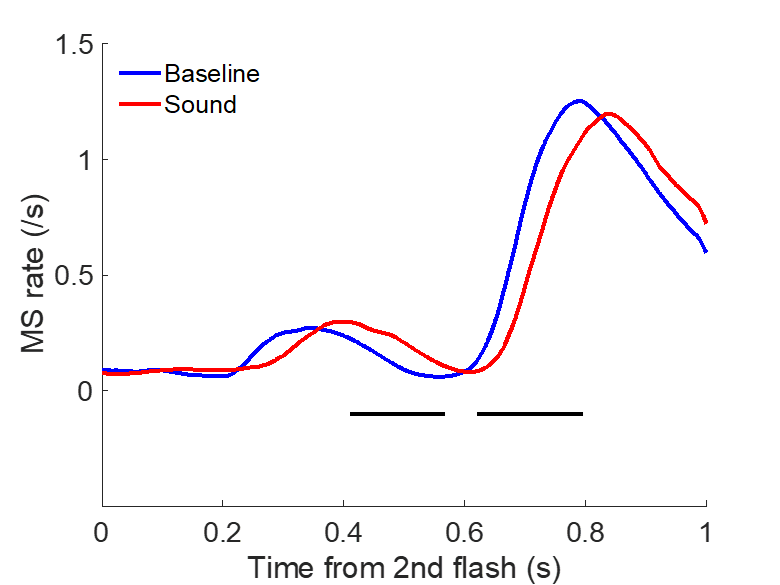


**Figure S12.** MS rate change time-locked to the onset of the second Ternus frame, parameterized with sound condition.


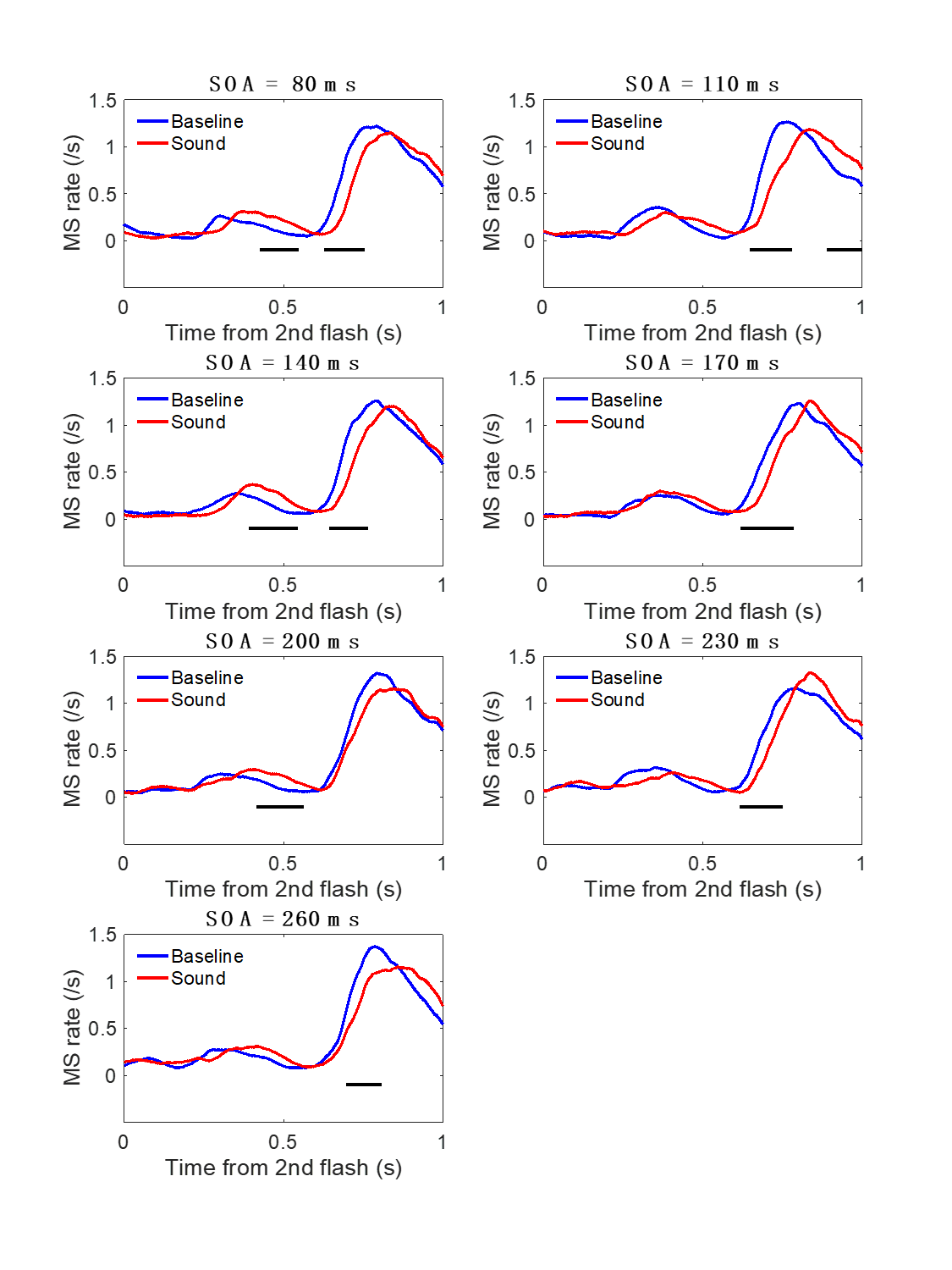


**Figure S13. MS results for main experiments**. MS rate as a function of the time from the second visual frame in both baseline (blue line) and sound (red line) conditions. The black horizontal lines indicate significant differences at the *p* < 0.05 level (cluster analysis).


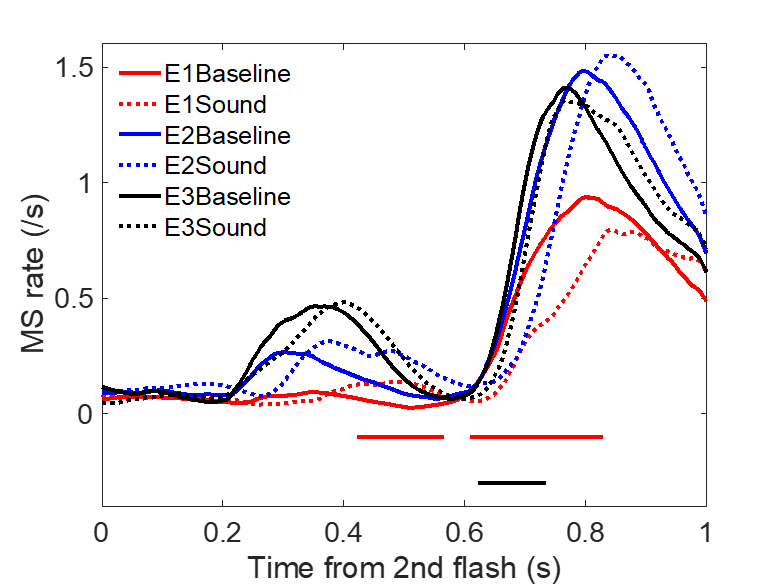


**Figure S14.** **Microsaccades (MS) and their time courses in baseline and sound conditions (time-locked to the 2^nd^ visual frame).** The horizontal coloured lines indicate significant differences at *p* < 0.05 in the cluster analysis and correspond to the colour code for each experiment.

**OMI in control Experiment (Exp.4) and Cross-Experiments comparison of OMI**

The mean MS rates for both the Ternus and localization tasks were 0.405 (±0.034) and 0.476 (±0.056) [*F*(1,67) = 1.147, *p* = 0.288, *η*^2^ = 0.017]. The two-way interaction between task and segment was significant [*F*(3,201) = 5.506, *p* = 0.001, *η*^2^ = 0.076]. The simple effects analysis indicated that for S1 the MS rates were lower in the Ternus task than in the localization task (*p* < 0.001), but no differences were found for S2-S4 (*p*s>0.5).

The main effect of sound conditions (baseline vs. sound) was significant, *F*(1,67)=4.742, *p*=0.033. The mean MS in baseline (0.456 ±0.034) was larger than the one in sound condition (0.425 ±0.033). The mean MSs across four segments were 0.268 ±0.024 (S1), 0.222 ±0.046 (S2), 0.311 ±0.035 (S3) and 0.962 ±0.078 (S4). The main effect of time segments was also significant, *F*(3,201)=64.126, *p*<0.001, *η*^2^ = 0.489.Bonferroni-corrected comparison showed that the MS in S4 was the largest, *p*s<0.001. No other 2-way or 3-way interaction effects have been found.

These findings suggest that the freezing effect in audiovisual integration was driven by OMI, with the critical time course of inhibition in earlier temporal range (S1) and rebound later (typically in S4).


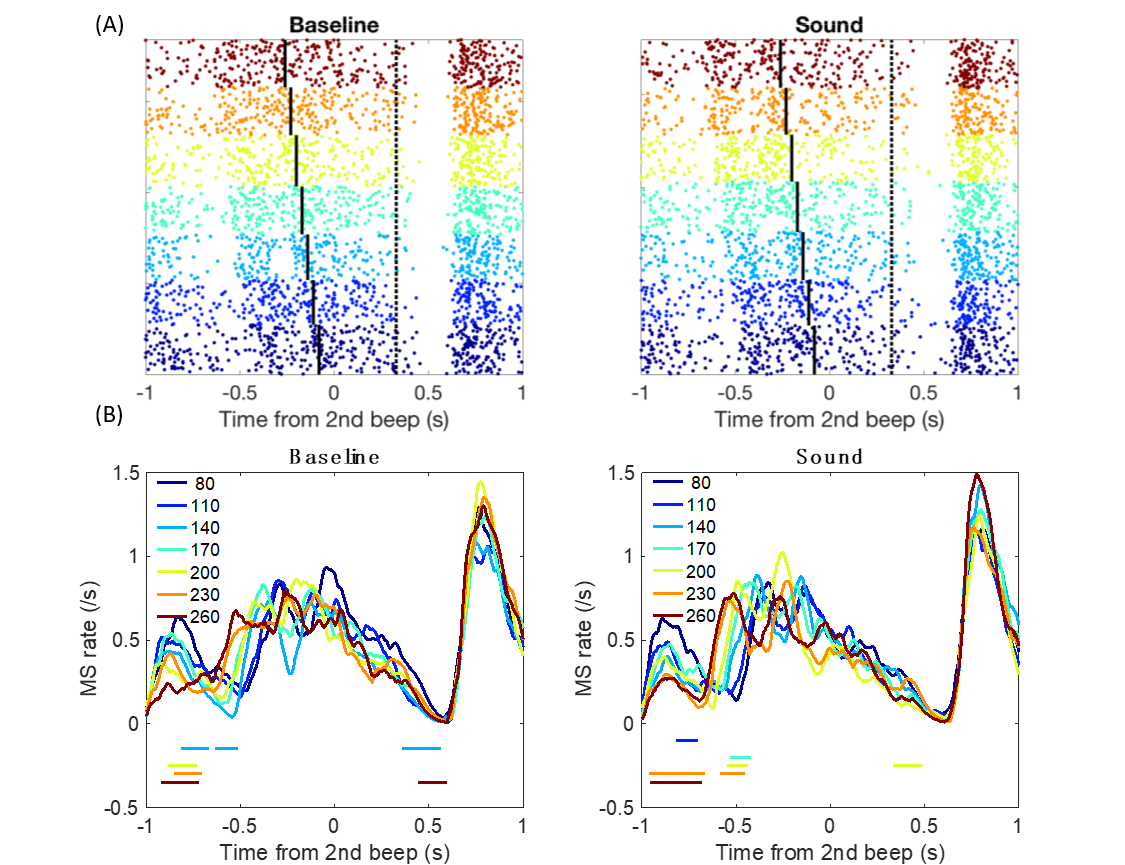


**Figure S15 (time-locked to 2^nd^ beep onset). MS rates in response to the visual localisation task with different SOAs, separated by sound conditions**. (A) Raster plots illustrate accumulated MS from all data. Each line represents one trial. Each dot represents a MS observed at the corresponding time point. Vertical black lines indicate the onset of 1^st^ beep. Vertical dotted lined indicate the onset of the question mark (for the participant to make a saccade to the peripheral question mark’s location). (B) MS rate change by time. Horizontal lines indicate significant clusters (p < 0.05) between each color-coded SOA condition and the SOA = 80 condition.


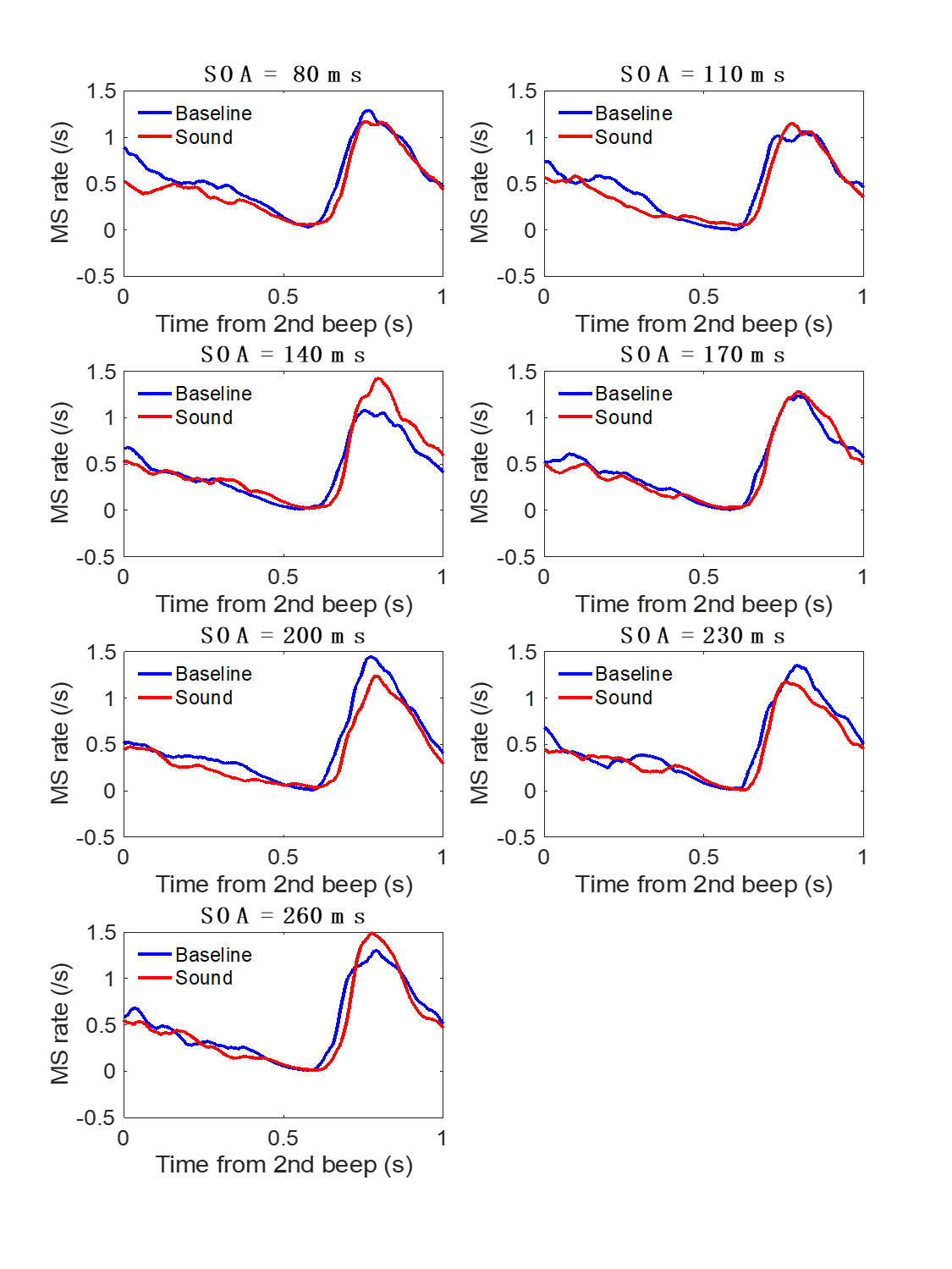


**Figure S16 MS rate in response to the visual localisation task as a function of the time from the second beep in both baseline (blue line) and sound (red line) conditions**. The black horizontal lines indicate significant clusters (p < 0.05) between baseline and sound conditions.


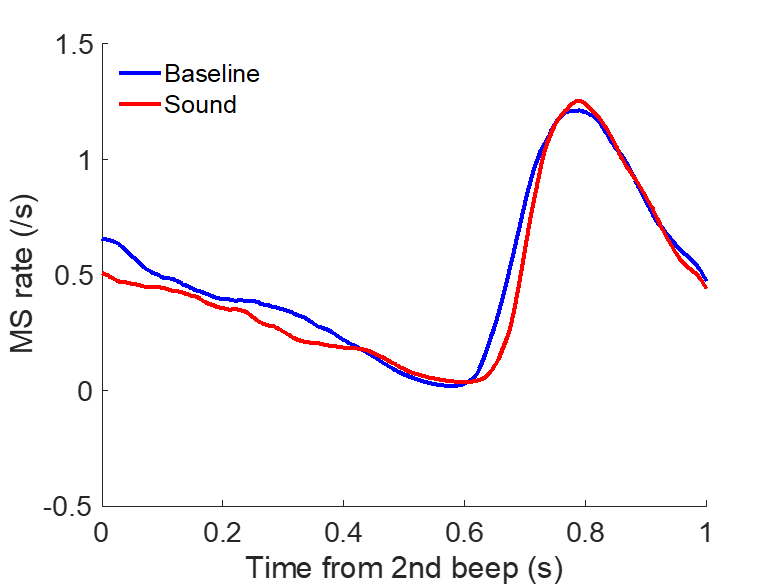


**Figure S17.** MS rate change time-locked to the onset of the second beep in localization task, parameterized with sound.
